# Supportive care interventions for men with urological cancers: a scoping review

**DOI:** 10.1007/s00520-023-07984-0

**Published:** 2023-08-21

**Authors:** Mohamad M. Saab, Megan McCarthy, Mike Murphy, Katarina Medved, Maria O’Malley, Richard M. Bambury, Jack P. Gleeson, Brendan Noonan

**Affiliations:** 1 Catherine McAuley School of Nursing and Midwifery, College of Medicine and Health, University College Cork, Cork, Ireland; 2 School of Applied Psychology, College of Arts, Celtic Studies & Social Sciences, University College Cork, Cork, Ireland; 3 Cancer Research @UCC, College of Medicine and Health, University College Cork, Cork, Ireland; 4 Medical Oncology Department, Cork University Hospital, Cork, Ireland

**Keywords:** Men, Needs assessment, Neoplasms, Prostatic neoplasms, Scoping review, Urologic neoplasms

## Abstract

**Purpose:**

To identify supportive care interventions for men with urological cancers.

**Methods:**

Experimental studies conducted among men with any urological cancer were eligible for inclusion. Academic Search Complete, CINAHL Plus with Full Text, MEDLINE, APA PsycArticles, APA PsycInfo, Social Sciences Full Text (H.W. Wilson), SocINDEX with Full Text, ERIC, Google Scholar and ClinicalTrials.gov were searched on 6 December 2022. No database limits were applied. The included studies were methodologically appraised. A narrative synthesis of the results was conducted.

**Results:**

Thirty studies were included with 10 categories of interventions identified. Over 300 outcomes were measured, and more than 100 instruments were used. Multicomponent interventions generally led to positive changes in physiological outcomes like body mass index, as well as exercise tolerance and quality of life. This change, however, was not sustained in the long term. Cognitive-behavioural interventions significantly improved psychological symptoms but seldom physical symptoms. Telephone and web-based interventions showed great promise in improving outcomes like depression, positive affect, negative affect, perceived stress, spiritual wellbeing and fatigue. Findings from physical activity/exercise-based interventions were promising for both, physical and psychological outcomes. Rehabilitative interventions were associated with significant improvements in quality of life, urinary symptoms and psychological symptoms, albeit in the short term. Mixed results were reported for nurse-led interventions, family-based interventions and nutritional interventions.

**Conclusion:**

All but one study focused exclusively on prostate cancer. The included studies were significantly heterogeneous. Multicomponent, cognitive-behavioural, telephone and web-based, physical activity/exercise-based and rehabilitative interventions showed great promise in improving various outcomes. This improvement, however, was often short-lived.

**Supplementary Information:**

The online version contains supplementary material available at 10.1007/s00520-023-07984-0.

## Introduction

Urological cancers comprise renal, ureteral, bladder, urethral, penile, prostate and testicular cancers [1]. Bladder cancer incidence and mortality rates are four times greater in men than in women and men are twice as likely to develop kidney cancer as women [2]. Prostate cancer (PCa) is the most common urological cancer, the most common cancer in men and the second leading cause of cancer mortality after lung cancer [2]. Testicular cancer is among the top five ranking cancers in men aged 15 to 44 years [2, 3].

New medical and surgical treatments are emerging for men with urological cancers, particularly PCa, leading to improved overall survival. However, men with PCa often have various unmet supportive care needs due to the cancer itself and/or its treatment [4]. In their systematic review of 17 studies, Paterson et al. classified the unmet needs of men living with and beyond PCa into social, spiritual, practical, daily living, patient-clinician communication, family-related, physical, psychological emotional, interpersonal/intimacy and health system/information needs [5]. From a psychological perspective, a systematic review of 117 observational studies found a high prevalence of significant depressive symptoms (17.07%), anxiety symptoms (16.86%) and suicidal ideation (9.85%) in men with PCa [6]. A further systematic review on support needs of men with PCa extracted themes of illness (including consistency and continuity of information, and designed with age, ethnicity and sexual orientation in mind), biographical (masculine identity, body changes, mortality, survival versus quality of life [QoL]) and everyday life (exercise, diet, relationships, stigma, support groups, work) [4]. Work and specialised support were raised specifically by younger and minority sexual orientation members respectively. Similar unmet needs were identified among men with testicular [7] and penile [8] cancers.

The well-documented unmet supportive care needs of men with urological cancers emphasize the urgency of appropriate and timely supportive care interventions. Such interventions are key to preventing and managing adverse effects of cancer and its treatment, reducing symptom and psychological burdens of urological cancers and enhancing men’s QoL across the continuum of the cancer journey [9, 10]. Supportive care for PCa has received much attention in systematic reviews published over the past decade. Such reviews either focused on a single supportive care intervention like exercise [11] and psychosexual care [12], identified rather than addressed the unmet supportive care needs of men with PCa [4–6], focused on men with low-risk localised PCa [13] or targeted specific symptoms like pain [14]. To the best of our knowledge, there have not been published reviews that pull all urinary cancers into one overarching scoping review and explore the different categories of interventions targeting men with various urinary cancers and supportive care needs. Therefore, the aim of this scoping review was to identify supportive care interventions for men with urological cancers. The review aimed to answer the following questions:

1. What supportive care interventions exist for men with urological cancers?
2. Which urological cancers were targeted in these interventions?
3. Which outcomes from these interventions were measured?
4. How were outcomes from these interventions measured?

## Methods

### Design

This scoping review was conducted according to the Joanna Briggs Institute methodology for scoping reviews [15] and reported using the Preferred Reporting Items for Systematic Reviews and Meta-Analyses extension for Scoping Reviews checklist [16].

### Eligibility criteria

The review inclusion and exclusion criteria were pre-determined using the population, intervention, comparison, outcome (PICO) framework [17] (Table 1). Given the broad scope of this review and to help maximise retrieval and reduce study selection and reporting bias, we included studies regardless of urinary cancer type, intervention type, comparator type, outcomes measured, year of publication, language of publication and geographical location.

**Table 1:** Eligibility criteria and search terms

PICO framework	Inclusion criteria	Exclusion criteria	Search terms
Population	Men of any age with a diagnosis of any primary or metastatic urological cancer; nonbinary and transgender individuals assigned male at birth; dyadic studies or studies with men who have different malignancies where findings from men with a urological cancer could be isolated.	Men with cancers other than urological cancers; women or individuals assigned female at birth; healthcare professionals	((uro* OR urin* OR peni* OR renal OR kidney* OR ureter* OR urethra* OR bladder OR testicular OR testis OR testes OR prostat* OR genitourinary OR genito-urinary OR “genito urinary”) N3 (cancer* OR tumour* OR tumor* OR neoplas* OR carcinoma OR malignan*)) (man OR men OR male OR males).
Intervention	Any supportive care intervention for men with urological cancers	Interventions targeting the prevention and early detection of urological cancers	((support* OR assist* OR help* OR aid* OR survivorship) N3 (interven* OR program* OR campaign* OR trial* OR experiment* OR educat*))
Comparison	Any pre-post comparison(s) within group (e.g. one-group pre-post) or between groups (e.g. intervention group[s] versus control group)	No comparator	Not specified
Outcome	Any patient outcome(s) as reported in the reviewed studies	Healthcare professional outcomes	Not specified

As aforementioned, urological cancer incidence and mortality are higher in men than women, and PCa is the most common urological cancer [2]. In addition, the supportive care needs of women with urological cancers like renal and bladder cancers are different from those of men [18, 19]. Therefore, only findings from men with a urological cancer were reported. This review was conducted to help identify outcomes and outcome measures for a future trial. The authors are aware of the broad literature in the area of urological cancers, particularly in the context of PCa. Therefore, only high-level evidence from randomised controlled trials (RCTs) and non-RCTs was included, and findings from underpowered feasibility and pilot studies were excluded. Qualitative, quantitative descriptive and literature review were also excluded due to the lack of an intervention and comparator.

### Information sources and search

Academic Search Complete, CINAHL Plus with Full Text, MEDLINE, APA PsycArticles, APA PsycInfo, Social Sciences Full Text (H.W. Wilson), SocINDEX with Full Text, ERIC, Google Scholar and ClinicalTrials.gov were searched on 6 December 2022. The reference lists of the included studies were hand searched for relevant studies. Keywords were combined using Boolean operators “AND” and “OR” and the proximity indicator “N.” Smart searching functions (i.e. phrase searching and truncation) were used. Subject headings for each database were identified, and a single search strategy was devised accordingly (Table 1).

### Selection of sources of evidence

Following the search, records were exported to Covidence online software, and duplicates were removed automatically [20]. Titles and abstracts were then screened, and irrelevant records were excluded. Full texts of the remaining records were retrieved and assessed further against the eligibility criteria. Reasons for exclusion of full-text records were recorded. Title, abstract and full-text screenings were conducted by two independent reviewers. Screening disagreements were resolved by a third reviewer.

### Data items and charting

Data from the included studies were extracted using a table adapted from two recent reviews [21, 22] under the following headings: reference, country, design, setting, sample size, cancer type, cancer stage, treatment received, dyad, type of intervention, person involved in intervention delivery, duration of intervention delivery, control group, frequency of outcome measurement, outcome measured, instrument/unit of measurement and results. Data were extracted by two reviewers and cross-checked for accuracy by the full review team. The full data extraction table is presented within Supplementary File 1.

### Critical appraisal

The included studies were methodologically appraised using the Mixed Methods Appraisal Tool (MMAT) which assesses the quality of five study categories namely qualitative studies, RCTs, non-RCTs, quantitative descriptive studies and mixed methods studies [23]. Voting for each of the quality items was conducted on a “Yes,” “No” and “Can’t tell” basis. The quality of the included RCTs and non-RCTs was appraised by one author and cross-checked by two authors. Conflicts in quality appraisal were resolved through consensus.

### Synthesis of results

Results were synthesised narratively according to the review aim and questions. First, the study characteristics were presented. This involved reporting on countries, designs, settings, sample sizes, cancer types, cancer stages, treatments received, whether studies were dyadic, types of interventions, follow-up times, outcomes measured and instruments used. Due to the significant heterogeneity within the included studies, it was not possible to pool results by outcome. Instead, results were synthesised and presented by intervention type. The categorisation of interventions was agreed by the full review team.

## Results

### Study selection

**Fig. 1:**
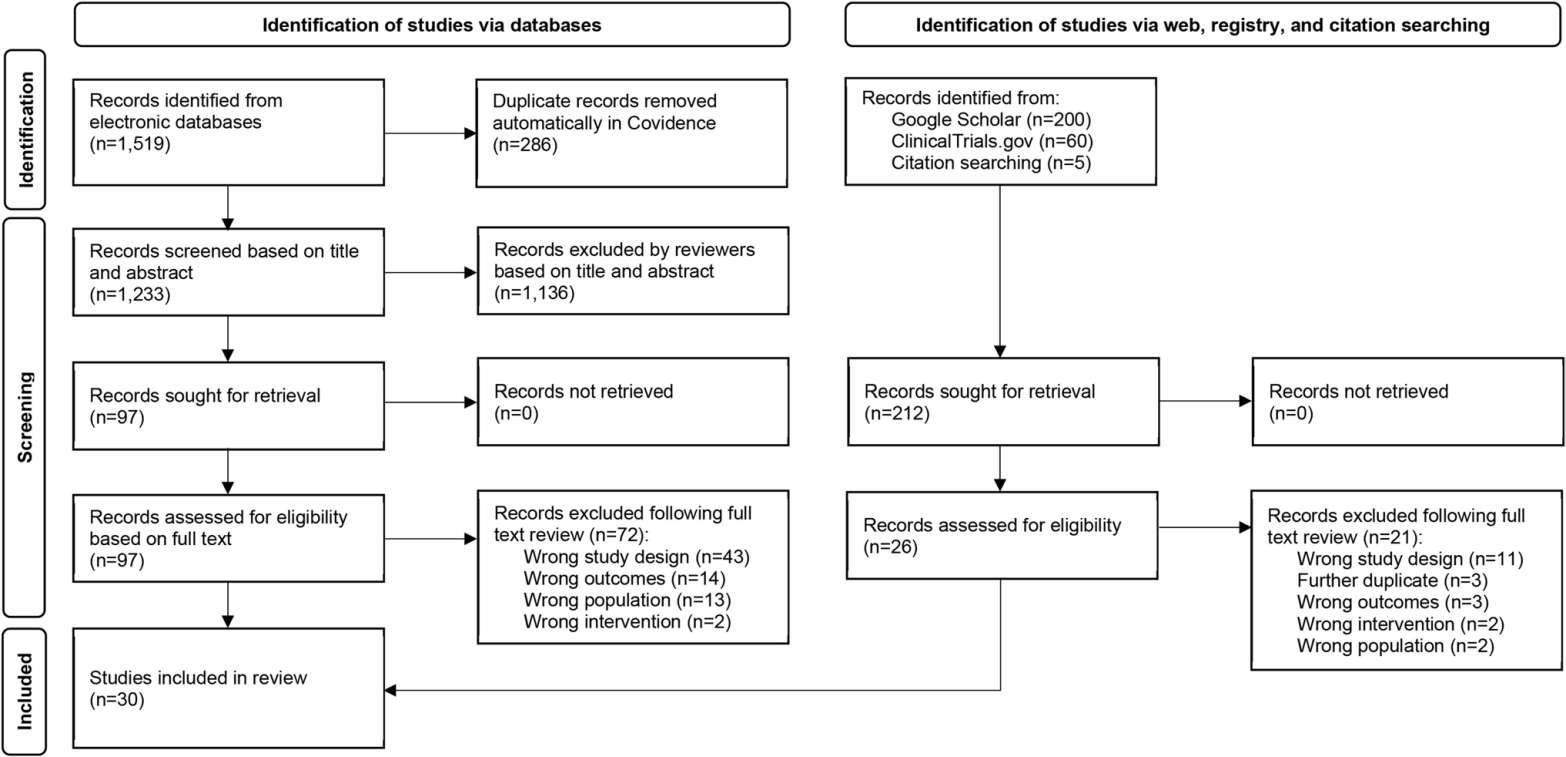
Study identification, screening and selection process

A total of 1519 records were identified from the databases. Following deletion of duplicates, the titles and abstracts of 1233 records were screened. Of those, 1136 irrelevant records were excluded. The full texts of the remaining 97 records were obtained and screened further, and 25 studies were included. Web, registry and citation searching yielded additional 265 records. Of those, 212 were retrieved, 26 were assessed for eligibility, and five were included for review. A total of 30 studies were included in this review. The full study selection process is presented in Fig. 1 [16].

### Study characteristics

The full study characteristics are presented in Table 2. About half of the included studies were conducted in the USA (*n* = 16) in acute care settings (*n* = 12). The majority were RCTs (*n* = 26) focused on PCa (*n* = 29) at varying stages (*n* = 12) or at early stage (*n* = 9). Treatments received varied and included, predominantly, androgen deprivation therapy together with prostatectomy and radiotherapy (*n* = 8), androgen deprivation therapy alone (*n* = 6) and androgen deprivation therapy with radiotherapy (*n* = 5). Eight studies were dyadic, including men with their partners. According to the review eligibility criteria, only data from men were extracted. Sample size ranged from 29 [24] to 859 [25] participants, and follow up time varied widely from 48 h [26] to 5 years [27].

**Table 2:** Study characteristics

Characteristic		*n*^a^
Country	USA	16
	Australia	5
	UK	3
	Denmark	2
	China	1
	Iran	1
	Sweden	1
	Switzerland	1
Design	Randomised controlled trial	26
	Non-randomised controlled trial	4
Setting	Hospital/medical centre/acute care	12
	Community	4
	Outpatient clinics	4
	Regional cancer centres, regional Veterans Affairs heath care centres, and cancer support groups	3
	Online	2
	Hospital and patient’s home	1
	Multiple health services	1
	Online and patient’s home	1
	Private clinics and hospitals	1
	Veterans Affairs	1
Sample size (min-max)	29-859	
Cancer type	Prostate cancer	29
	Testicular cancer	1
Cancer stage	Various stages	12
	Early stage	9
	Unclear	6
	Advanced stage	3
Treatment received	Androgen deprivation therapy, prostatectomy, and radiotherapy	8
	Androgen deprivation therapy	6
	Androgen deprivation therapy and radiotherapy	5
	Radiotherapy (external beam and/or brachytherapy)	3
	Prostatectomy	2
	Androgen deprivation therapy and prostatectomy	1
	Androgen deprivation therapy, chemotherapy, prostatectomy, radiotherapy, and watchful waiting	1
	Chemotherapy and radiotherapy	1
	Prostatectomy and radiotherapy	1
	Not reported	2
Dyad (yes/no)	No	22
	Yes	8
Type of intervention	Multicomponent interventions	7
	Psychological/ behavioural interventions	6
	Telephone and web-based interventions	4
	Physical activity/exercise-based interventions	3
	Rehabilitation interventions	3
	Nurse-led interventions	2
	Psycho-sexual interventions	2
	Family-based interventions	1
	Information support interventions	1
	Nutritional interventions	1
Follow-up time (min-max)	48 h-5 years	
Outcome measured^b^	Physical symptoms	72
	Physical health and wellbeing	61
	Mental and emotional health	42
	Psychological symptoms	29
	Sexuality and sexual health	27
	Social support and relationships	19
	Information and knowledge acquisition	18
	Quality of life	14
	Self-efficacy and confidence	13
	Health perceptions and beliefs	8
	Others	18
Instrument^c^	Expanded Prostate Cancer Index Composite	10
	Short Form Survey	8
	European Organization for the Research and Treatment of Cancer Quality of Life Questionnaires	7
	Center for Epidemiologic Studies-Depression Scale	5

^a^*n* = 30 studies unless otherwise indicated

^b^*n* = 321 outcomes measured. The full outcomes are listed in Supplemental File 2

^c^*n* = 112 instruments used. The most commonly used instruments are listed here. The full list of instruments is enclosed within Supplemental File 3

Ten categories of interventions were identified including the following: multicomponent interventions (*n* = 7), psychological/behavioural interventions (*n* = 6), telephone and web-based interventions (*n* = 4), physical activity/exercise-based interventions (*n* = 3), rehabilitation interventions (*n* = 3), nurse-led interventions (*n* = 2), psychosexual interventions (*n* = 2), family-based interventions (*n* = 1), information support interventions (*n* = 1) and nutritional interventions (*n* = 1). A detailed description of those is enclosed within Supplementary File 1.

A total of 321 different outcomes were measured. These were categorised into the following 11 categories: physical symptoms (*n* = 72), physical health and wellbeing (*n* = 61), mental and emotional health (*n* = 42), psychological symptoms (*n* = 29), sexuality and sexual health (*n* = 27), social support and relationships (*n* = 19), information and knowledge acquisition (*n* = 18), QoL (*n* = 14), self-efficacy and confidence (*n* = 13), health perceptions and beliefs (*n* = 8) and others (*n* = 18). A further breakdown of these categories can be found in Supplementary File 2.

As for outcome measures, 112 different instruments were used. The most used instrument was Expanded Prostate Cancer Index Composite (EPIC, *n* = 10), followed by the Short Form Survey (*n* = 8), the European Organization for the Research and Treatment of Cancer Quality of Life Questionnaires (EORTC QLQ, *n* = 7) and the Center for Epidemiologic Studies-Depression Scale (*n* = 5). The full list of instruments is enclosed within Supplementary File 3.

### Critical appraisal

Two study categories were assessed for methodological quality: RCTs (*n* = 26) and non-RCTs (*n* = 4), both with seven quality appraisal items (Table 3). All studies had clear aims, adequately addressed with the data collected. All RCTs had comparable groups at baseline, with complete data outcomes (*n* = 22) and adherence to the assigned intervention (*n* = 24) for the majority. Only nine RCTs reported blinding the outcome assessor [26, 28–35]. Regarding non-RCTs (*n* = 4), the representativeness of the target population was adequately reported in all but one non-RCT [36]. All non-RCTs used appropriate measures, with two studies clearly reporting on complete outcome data [25, 37], and three studies reporting on confounding factors [24, 25, 37]

**Table 3:** Quality assessment of the included studies (*n* = 30)

Study designs	Author(s) and year	Quality appraisal items^a^											
		1	2	3	4	5	6	7	8	9	10	11	12
Randomised controlled trials^b^	Badger et al. (2011)	Y	Y	CT	Y	Y	CT	Y					
	Badger et al. (2013)	Y	Y	CT	Y	Y	CT	Y					
	Bouchard et al. (2019)	Y	Y	Y	Y	Y	N	Y					
	Bourke et al. (2014)	Y	Y	Y	Y	Y	Y	Y					
	Carmack Taylor et al. (2006)	Y	Y	Y	Y	Y	Y	Y					
	Carmack Taylor et al. (2007)	Y	Y	Y	Y	Y	CT	Y					
	Chambers et al. (2019)	Y	Y	CT	Y	Y	CT	Y					
	Cohen et al. (2011)	Y	Y	Y	Y	Y	Y	Y					
	Dieperink et al. (2013)	Y	Y	Y	Y	Y	Y	Y					
	Dieperink et al. (2017)	Y	Y	Y	Y	Y	CT	Y					
	Faithfull et al. (2022)	Y	Y	Y	Y	Y	N	Y					
	Forslund et al. (2020)	Y	Y	Y	Y	N	CT	Y					
	Giesler et al. (2005)	Y	Y	Y	Y	Y	Y	Y					
	Goode et al. (2022)	Y	Y	Y	Y	Y	N	Y					
	Mardani et al. (2021)	Y	Y	Y	Y	Y	N	Y					
	Moynihan et al. (1998)	Y	Y	Y	Y	Y	Y	Y					
	Northouse et al. (2007)	Y	Y	CT	Y	Y	Y	Y					
	Penedo et al. (2020)	Y	Y	Y	Y	Y	CT	Y					
	Segrin et al. (2012)	Y	Y	CT	Y	Y	CT	Y					
	Skolarus et al. (2019)	Y	Y	Y	Y	Y	CT	Y					
	Tagai et al. (2021)	Y	Y	Y	Y	N	CT	Y					
	Winters-Stone et al. (2016)	Y	Y	Y	Y	Y	Y	Y					
	Wittmann et al. (2022)	Y	Y	N	Y	N	CT	N					
	Wootten et al. (2015)	Y	Y	Y	Y	N	CT	N					
	Yang et al. (2021)	Y	Y	Y	Y	Y	CT	Y					
	Zhang et al. (2015)	Y	Y	Y	Y	Y	Y	Y					
Non-randomised studies^c^	Beydoun et al. (2014)	Y	Y						Y	Y	Y	Y	Y
	Leahy et al. (2013)	Y	Y						Y	Y	Y	Y	Y
	Mareschal et al. (2017)	Y	Y						Y	Y	N	Y	Y
	Yates et al. (2022)	Y	Y						N	Y	N	N	N

*CT* Can’t tell, *N* No, *Y* Yes

^a^All studies: 1 = Clear research questions/aims, 2 = Data collected address research question/aims

^b^Randomised controlled trials: 3 = Randomisation appropriately performed, 4 = Groups comparable at baseline, 5 = There are complete outcome data, 6 = Outcome assessors blinded to the intervention, 7 = Participants adhered to the assigned intervention

^c^Non-randomised studies: 8 = Participants representative of target population, 9 = Measurements appropriate regarding both the outcome and the intervention, 10 = Complete outcome data, 11 = Confounders accounted for in the design and analysis, 12 = The intervention administered as intended

### Synthesis of results

Results from the included studies were synthesised narratively by intervention type as follows: multicomponent interventions, psychological/behavioural interventions, telephone and web-based interventions, physical activity/exercise-based interventions, rehabilitation interventions, nurse-led interventions, psychosexual interventions, family-based interventions, information support interventions and nutritional interventions. Findings from individual studies are summarised and represented visually in Table 4. The full results are presented in Supplementary File 1.

**Table 4:** Summary of findings table (*n* = 30)

Intervention	Reference	Design	Sample (n)	Cancer	Outcomes										
					Physical symptoms	Physical health and wellbeing	Mental and emotional health	Sexuality and sexual health	Psychological symptoms	Self-efficacy and confidence	Information and knowledge acquisition	Social support and relationships	Quality of life	Health perceptions and beliefs	Others
Multicomponent	Bourke et al. (2014)	RCT	68	Prostate	↑	↕	–	–	–	–	–	–	↓	–	↔ Serum PSA
	Carmack Taylor et al. (2006)	RCT	113	Prostate	↔	↕	↔	–	↔	↓	–	↔	–	↔	–
	Carmack Taylor et al. (2007)	RCT	113	Prostate	↕	↕	↔	–	↓	↔	–	↕	–	↔	–
	Goode et al. (2022)	RCT	245	Prostate	↔	–	–	–	–	–	–	–	↔	↔	↔ Satisfaction, Resumption of normal activities Return to work
	Mareschal et al. (2017)	Non-RCT	29	Prostate	↔	↔	↕	↔	↔	↔	–	↔	–	↔	↔ PSA ↑ Cancer control
	Yates et al. (2022)	Non-RCT	136	Prostate	↔	–	–	↔	–	–	–	–	–	–	–
	Zhang et al. (2015)	RCT	244	Prostate	↕	–	–	–	–	–	–	–	–	–	–
Psychological/ behavioural	Bouchard et al. (2019)	RCT	154	Prostate	–	–	–	–	↑	–	–	–	–	–	–
	Cohen et al. (2011)	RCT	127	Prostate	–	–	–	–	↑	–	–	–	–	–	–
	Moynihan et al. (1998)	RCT	141	Testes	↑	–	↔	↔	↔	–	–	↔	–	–	↔ Healthcare orientation Vocational environment
	Penedo et al. (2020)	RCT	154	Prostate	↔	–	↑	–	↑	↑	–	–	↔	–	–
	Skolarus et al. (2019)	RCT	556	Prostate	↔	–	↕	↔	–	↕	–	–	↕	↔	↔ Cancer control
	Wootten et al. (2015)	RCT	104	Prostate	–	–	↕	–	↑	↔	–	–	–	–	↑ Informed decision
Telephone/ web-based	Badger et al. (2011)	RCT	70	Prostate	↑	–	↕	–	↑	–	–	↕	–	–	–
	Badger et al. (2013)	RCT	64	Prostate	–	–	↕	–	↕	–	↔	↑	–	–	–
	Segrin et al. (2012)	RCT	63	Prostate	↑	–	↑	–	↑	–	–	–	–	–	–
	Tagai et al. (2021)	RCT	210	Prostate	↕	–	↑	↑	–	↔	–	↔	–	–	↔ Planning
Exercise/ physical	Beydoun et al. (2014)	Non-RCT	859	Prostate	–	↕	–	–	–	–	–	–	–	–	–
	Mardani et al. (2021)	RCT	71	Prostate	↕	↑	↓	↕	–	–	–	↓	↔	–	↔ Financial difficulties
	Winters Stone et al. (2016)	RCT	64	Prostate	–	↕	↔	–	–	–	–	–	–	–	–
Rehab	Dieperink et al. (2013)	RCT	161	Prostate	↕	–	–	↔	–	–	–	–	↕	–	↔ Digital evaluation
	Dieperink et al. (2017)	RCT	161	Prostate	–	–	↕	–	↔	–	–	–	–	–	–
	Faithfull et al. (2022)	RCT	56	Prostate	↕	–	–	–	↔	↕	–	–	↔	–	–
Nurse-led support	Giesler et al. (2005)	RCT	99	Prostate	↔	↕	↕	↕	↕	–	–	↔	–	↔	–
	Leahy et al. (2012)	Non-RCT	169	Prostate	↔	–	–	↔	↔	–	–	–	–	–	↑ Patient satisfaction (low risk) ↔ Patient satisfaction (high risk)
Psycho- sexual	Chambers et al. (2019)	RCT	107	Prostate	–	–	–	↕	–	↑	–	–	–	–	–
	Wittman et al. (2022)	RCT	192	Prostate	–	–	–	↕	–	–	–	–	–	–	–
Family-based	Northouse et al. (2007)	RCT	218	Prostate	↔	–	↕	↔	–	↔	–	–	↔	–	↓ Communication ↔ Appraisal of illness
Information support	Yang et al. (2021)	RCT	95	Prostate	↔	↕	–	↑	↔	↓	↕	–	↔	↓	↓ Self-decision making ↔ Self-decompression and PSA
Nutritional	Forslund et al. (2019)	RCT	180	Prostate	↕	–	–	–	–	–	–	–	↔	–	–

*PSA* Prostate specific antigen, *RCT *Randomised controlled trial ↑ statistically significant improvement, ↓ statistically significant deterioration, ↔ no statistically significant difference, ↕ mixed results, – not reported/measured

#### Multicomponent interventions

Seven studies reported on the effect of multicomponent interventions [24, 28, 29, 35, 36, 38, 39]. The Active for Life intervention comprises two programs, the lifestyle program (cognitive-behavioural curriculum focused on increasing physical activity) and the educational support program (facilitated discussion and expert speaker covering topics such as sexuality, treatment side effects or diet). This intervention was tested in two studies [29, 39]. In the first study, the only significant improvement was noted in the processes of change for physical activity at 6 months, whereby the mean score on the Processes of Change for Physical Activity Questionnaire was 4.5/5 in the lifestyle group, 4/5 in the educational group and 3.7/5 in the control group (p = 0.02). This change, however, was not maintained at 12 months [29]. In their second study, Carmack Taylor et al. found that, in comparison to the control group, the intervention groups combined reported less bodily pain (p = 0.04) at 12 months but not at 6 months, and participants in both intervention groups reported less pain (p = 0.002) at 6 months but not at 12 months [39]. Anxiety was significantly reduced in both intervention groups at 6 months (p = 0.03) but not at 12 months. Depression decreased significantly for both intervention groups at 6 months (p = 0.03) and 12 months (p = 0.006) [39].

Combined tapered exercise and dietary advice with integrated behaviour change support were associated with significant improvements in patient-reported fatigue measured using the Functional Assessment of Cancer Therapy-Fatigue Questionnaire (mean in the intervention group 45.8/52 vs 42.4/52 in the control group), total exercise behaviour measured using the Godin Leisure Score Index (mean difference 14.6 in the intervention group vs the control group) and aerobic exercise tolerance measured using the Symptom-Limited Graded Exercise Test (mean difference 121.2 in the intervention group vs the control group), 12 weeks (*p* < 0.001 for all three outcomes) and 6 months (*p* < 0.007, *p* = 0.038, *p* < 0.001 respectively) post-test [28]. Dietary behaviour (reduced total fat intake) measured using 3-day diet diaries (mean difference − 11.1 in the intervention group vs the control group) and disease-specific QoL assessed using the Functional Assessment of Cancer Therapy-Prostate Questionnaire (mean difference 8.9 points in the intervention group vs the control group) improved significantly at 12 weeks (*p* = 0.039 and *p* = 0.001 respectively). The improvement in QoL was not sustained at 6 months [28]. A similar intervention comprising nutritional, physical and psychological coaching for men with different urological cancers was associated with significantly decreased health worries (*p* = 0.028), whereby the mean score decreased significantly from 20.8/100 at baseline to 14.4/100 at 12 months. Improved cancer control was also reported whereby the mean score increased from 62.7/100 at baseline to 72.7/100 12 months post-test (*p* = 0.046) [24]. Most outcomes, however, did not reach statistical significance.

The Stay Dry program involved teaching patients pelvic floor muscle exercise and self-management skills [35]. Based on participant diary data, the biofeedback plus support and biofeedback plus telephone groups had a significantly lower daily urinary leakage frequency than the control group at 3 months (mean number of leakages/day in biofeedback plus support 1.6 vs 1.9 for biofeedback plus telephone vs 2.7 for the control group, *p*≤0.05), but not at 6 months. The biofeedback plus support group but not the biofeedback plus telephone group had 13.3 g lower leakage at 6 months in comparison to the control group (*p* = 0.003). At 6 months, the biofeedback plus telephone and biofeedback plus support groups reported lower symptom severity (*p* = 0.001) and fewer incontinence problems (*p* = 0.01) than the control group [35]. A similar study found that patient satisfaction improved significantly at 6 months, whereby 60% of participants were “somewhat satisfied” in the intervention group in comparison to 40% in the control group (*p* = 0.04) [38].

#### Psychological/behavioural interventions

Six studies assessed psychological/behavioural interventions [26, 32, 40–43]. Of those, three used cognitive-behavioural interventions [40, 41, 43]. Generally, these interventions significantly improved psychological symptoms but seldom physical symptoms. A 10-week cognitive behavioural stress management intervention was associated with a significant improvement in anxiety at 6 months (intervention group score 10.17/54 vs 8.27/54 for the control group using the Memorial Anxiety Scale for Prostate Cancer, *p* < 0.001) and 12 months (intervention group score 9.03/54 vs 8.94/54 for the control group, *p* = 0.011) [40]. Another study also found that a 10-week cognitive-behavioural stress and self-management skills with relaxation skills training program was associated with improved stress management skills and self-efficacy (*p* = 0.004) and reduced cancer-related anxiety (*p* = 0.023) and fear of reoccurrence (*p* = 0.010) [41]. Similarly, a 10-week psychological online intervention was associated with a significant increase in informed decision (p = 0.035) and outlook (*p* = 0.02) and a decrease in regret (*p* = 0.047) and psychological distress (p = 0.02) [43]. Another behavioural intervention in the form of interactive voice response symptom management and tailored newsletters over 3 months led to significant improvement in confidence in symptom-self management at 12 months (mean EPIC score in the intervention group 13.5/15 vs 12.9/15 in the control group, *p* = 0.03), but not at 5 months [42].

Moynihan et al. [32] found that 8 weeks of adjuvant psychological therapy compared to usual care among patients with testicular cancer were associated with significant improvements in physical symptoms measured using the Rotterdam Symptom Checklist (mean difference − 2.3 intervention vs control, *p* < 0.01) and psychological adjustment (vocational environment) (mean difference 2.9 intervention vs control, *p* < 0.05). Another study tested the effect of stress management (individual sessions with a clinical psychologist and a stress management guide) and supportive attention (individual sessions with a clinical psychologist) [26]. Using the Profile of Mood States Questionnaire, patients in the stress management intervention were found to have significantly lower mood disturbance in comparison to standard care (mean 8.2/18 in the intervention group vs 11.9/18 in the control group, *p* = 0.006).

#### Telephone and web-based interventions

Telephone-based health education and counselling were tested in four studies [44–47]. Badger et al. tested the effect of the telephone-based health education attention condition and the telephone interpersonal counselling [44]. They found that depression, positive affect, negative affect, perceived stress, spiritual wellbeing and fatigue improved significantly at least 8 weeks post-test, favouring health education attention condition (all *p* < 0.001). Badger et al. then compared the effect of the telephone interpersonal counselling versus telephone health education [45]. Telephone health education was associated with more favourable depression outcomes (*p*≤0.05) while telephone interpersonal counselling was associated with improved positive affect (*p*≤0.05).

In keeping with virtually delivered interventions, a web-based intervention aimed to improve adaptive coping among patients with PCa was associated with significant improvements in urinary health and urinary incontinence measured using the 4-item Urinary Incontinence Scale (*p* < 0.001 for intervention and control groups) as well as sexual dysfunction and general coping techniques measured using the 7-item Interpersonal Coping Scale (*p* < 0.05 for both groups) [46]. While practical concerns increased in both groups (*p* < 0.001), the intervention group had less of an increase than the control group at 6 months (mean score in the intervention group 1.58/5 vs 1.68/5 in the control group, *p* < 0.05).

#### Physical activity/exercise-based interventions

Three papers studied the effect of exercise and physical activity interventions [25, 34, 48]. Of those, two were promising [25, 48]. Beydoun et al. tested the effect of an exercise intervention with three interventional streams including group (face-to-face) exercise sessions, home-based exercise, or a support programme for those unable to exercise [25]. Ten weeks post-test, participants in the group stream had significantly smaller waist (mean reduction from 105.2 to 103.5 cm, *p*≤0.0001) and hip circumference (mean reduction from 106.5 to 105.5 cm, *p* = 0.015), completion of a 400-m walk (mean time decreased from 304 to 271 s, *p*≤0.0001), reduction in systolic (mean difference − 3.4, *p* = 0.0044) and diastolic (mean difference − 3.1, *p*≤0.0001) blood pressure and improved strength (*p*≤0.0001) [25]. Compared to baseline, a 12-week programme comprising aerobic, resistance, flexible and pelvic floor muscle exercises was associated with significant improvement in several QoL domains as measured using the EORTC QLQ-C30 and EORTC QLQ-Prostate Cancer Module (QLQ-PR25). These included physical function (*p* < 0.001), role function (*p* < 0.001), emotional function (*p* < 0.001), social function (*p* < 0.001), fatigue (*p* < 0.001), insomnia (*p* < 0.001), constipation (*p* = 0.03), diarrhoea (*p* = 0.005), sexual function (*p* = 0.01), urinary symptoms (*p* < 0.001), bowel symptoms (*p* < 0.001) and hormonal treatment–related symptoms (*p* = 0.001) [48]. Winters Stone et al. tested the effect of the exercising together project providing strength training twice weekly for 6 months. The only significant improvement was noted in bench press (*p* < 0.01) and physical activity (*p* < 0.01) [34].

#### Rehabilitation interventions

Three studies reported on the effect of rehabilitation intervention [30, 49, 50]. Of those, two tested the effect of the same multidisciplinary rehabilitation programme involving nursing counselling sessions and instructive sessions with a physical therapist [30, 49]. At 6 months, the programme was associated with significant improvements in physical QoL (*p* = 0.002), urinary (sum score) (mean difference in the intervention group 13.3 vs 9 in the control group, *p* = 0.023), urinary irritative symptoms (mean difference in the intervention group 17.6 vs 11.6 in the control group, *p* = 0.011) and hormonal symptoms (mean difference in the intervention group 2.3 vs − 2.8 in the control group, *p* = 0.018), all measured using EPIC [30]. In their second study, Dieperink et al. found that the intervention was associated with a significant improvement in fighting spirit whereby the mean scores on the Mini-Mental Adjustment to Cancer Scale increased slightly from 12.0 to 12.1 points (*p* = 0.025) at 6 months but not at 3 years [49]. Cognitive avoidance, on the other hand, improved at 3 years (*p* = 0.044) with mean scores decreasing in the intervention group from 9.6/16 at baseline to 9.3/16 at 3 years. This improvement was not evident at 6 months. The remaining study tested the effect of the rehabilitative pathway (group support, education and pelvic floor muscle exercises) [50]. This study found that voiding symptoms measured using the International Continence Scale improved by − 1.9 points in the intervention group and by − 0.8 points in the control group at 6 months (*p* = 0.017). Incontinence symptoms measured using the same scale improved by − 0.9 points in the intervention group and deteriorated by 0.5 points in the control group (*p* = 0.029). Self-efficacy for managing symptoms measured using the Self-Efficacy for Symptom Control Inventory also improved significantly at 3 months, with an increase of 11 points in the intervention group and a decrease of 5.9 points in the control group (*p* = 0.017) [50].

#### Nurse-led interventions

Two studies reported on nurse-led interventions [31, 37]. Leahy et al. found that nurse-led telephone consultations for 6 months did not lead to significant improvements in outcomes [37]. Similarly, following a nurse-led cancer care intervention, statistical significance was not reached for most outcomes [31]. Other outcomes did not improve significantly until 4, 7 and/or 12 months post-test. These included sexual limitation (*p* = 0.05 at 7 months and *p* = 0.02 at 12 months), cancer worry (*p* = 0.03 at 12 months), depression and urinary bother (*p* = 0.015 at 4 months and *p* = 0.007 at 7 months), depression and physical role function (*p* = 0.014 at 12 months) and physical role (*p* = 0.014 at 12 months) [31].

#### Psychosexual interventions

Psychosexual interventions were tested in two studies [27, 51]. Chambers et al. tested the effect of two interventions including a peer support group and nurse counselling group [27]. Five years post-test, sexual self-confidence improved significantly in the control group in comparison to both intervention groups (*p* = 0.043). The control group had less sexual supportive care needs than the peer support group (*p* = 0.001) and the nurse counselling group (*p* = 0.01). Masculine self-esteem improved significantly at 5 years in the nurse counselling group (*p* = 0.045). In comparison to the control group, the overall use of treatments for sexual problems increased significantly in the peer support group whereby 80% of participants used these treatments at 3 years versus 86.84% at 4 years and 5 years (*p* = 0.040 at 3 years, *p* = 0.002 at 4 years, *p* = 0.005 at 5 years). Similarly, the overall use of treatments for sexual problems increased significantly in the nurse counselling group whereby 79.07% of participants used these treatments at 4 years versus 80.49% at 5 years (*p* = 0.004 at 4 years, *p* = 0.007 at 5 years) [27]. The second study tested the effect of a sexual recovery intervention. In comparison to baseline, 73% of participants in the intervention group reported an increase in sexual activity compared to 60% in the control group at 3 months (*p* = 0.037). Increased vaginal penetration was also reported more often by the intervention group (13%) in comparison to the control group (5%) at 3 months (*p* = 0.008) but not 6 months [51]. No other outcomes reached statistical significance.

#### Family-based interventions

Northouse et al. tested the effect of a family-based intervention [33]. Uncertainty measured using the 28-item Mishel Uncertainty in Illness Scale (mean in the intervention group 56.9/140 vs 60/140 in the control group, *p* = 0.03) and communication measured using the 32-item Lewis Mutuality and Interpersonal Sensitivity Scale (mean in the intervention group 3.80/5 vs 3.69/5 in the control group *p* = 0.03) improved significantly at 4 months but not at 12 months. Otherwise, most outcomes did not reach statistical significance.

#### Information support interventions

One study reported on the effect of an information support programme on more than 30 outcomes [52]. Of those, 24 improved significantly (all *p* < 0.05). These can be broadly categorised into information acquisition, information assistance, disease knowledge mastery, healthy lifestyle, self-efficacy, positive attitude, self-decision making and healthy behaviour adherence [52].

#### Nutritional interventions

One study reported on the effect of a nutritional intervention comprising three sessions with a dietician before, 4 weeks after and 8 weeks after radiotherapy [53]. Results from the Gastrointestinal Side Effects Questionnaire indicate that the intervention was associated with significantly less blood in the stools (mean in the intervention group 0/10 vs 1/10 in the control group, *p* = 0.047) and significantly reduced flatulence (mean in the intervention group 2/10 vs 3/10 in the control group, *p* = 0.014). Otherwise, most outcomes did not reach statistical significance.

## Discussion

Thirty studies were included in this scoping review. All but one study focused exclusively on PCa [32]. A total of 321 outcomes were measured with physical symptoms, physical health and wellbeing and mental and emotional health ranking top 3. One hundred and twelve different instruments were used with EPIC, Short Form Survey and the EORTC QLQ ranking top 3. Ten categories of interventions were identified. Multicomponent interventions led to positive yet short-term improvements in various physiological outcomes, exercise tolerance and QoL. Cognitive-behavioural interventions significantly improved psychological symptoms but seldom physical symptoms. Outcomes like depression, positive affect, negative affect, perceived stress, spiritual wellbeing and fatigue improved following telephone and web-based interventions. Findings from physical activity/exercise-based interventions were promising for both, physical and psychological outcomes. Rehabilitative interventions were associated with improved QoL, urinary symptoms and psychological symptoms, albeit in the short term. Mixed results were reported for family-based, psychosexual, information-support, nurse-led and nutritional interventions.

Most studies included in this review focused on PCa, which was not unexpected given the high incidence of PCa globally [2]. Men living with and beyond PCa have unmet social, physical and psychological needs [5, 6]. Notwithstanding the dominant focus on PCa, we identified a wide range of supportive care interventions available for men, including multicomponent, psychological, physical activity, nurse-led and family-based interventions. The large number of outcomes measured across the included studies was surprising and necessitated their grouping into different categories. Supportive care interventions focusing on the physical and psychological wellbeing of men were by far the greatest in number. This is not surprising, given that men with urological cancers have consistently reported unmet needs in these areas regardless of disease stage and treatment(s) received [54].

Across the 30 reviewed studies, over 100 different instruments were used to measure outcomes. This highlights the complexity of supportive care needs to be addressed in this cohort of men [4, 7]. It is important to note that the critical appraisal of included studies revealed positive results even though the majority of RCTs did not make it evident that assessors were blinded to the intervention. We know that blinding of outcome assessors/study investigators lowers the risk of detection bias; however, we acknowledge that blinding is not always feasible, particularly in the reviewed studies.

Across the multicomponent intervention studies, several outcomes did not reach statistical significance. The most recent study by Goode et al. [38] involved a mobile telehealth program for post-prostatectomy intervention similar in design to the Stay Dry Program study by Zhang et al. [35] which was not associated with significant improvement in key areas such as QoL, incontinence, resumption of activities and return to work. Goode et al. recommend that future study designs need to consider comparing in-clinic behavioural programs to no treatment or usual care [38]. Psychological/behavioural interventions in general significantly improved psychological symptoms but seldom physical symptoms. Bouchard et al.’s cognitive behavioural stress management intervention is one such example associated with a significant improvement in anxiety amongst racially diverse PCa patients at 6 and 12 months when compared to a health promotion control alone [40]. A particular strength of this study was its large proportion of Black participants, higher than previously published psychosocial PCa research.

Virtually delivered interventions (telephone and web-based) demonstrated, in general, a potential to improve certain aspects of adaptive coping among survivors but struggled to adequately address psychosocial domains [46]. Further research is needed to better understand the gaps in virtually delivered interventions contributing to low engagement and limited improvement across all domains of functional QoL and adaptive coping [46]. In their RCT exploring the effect of exercise on the QoL of PCa survivors, Mardani et al. reported significant improvements in physical, emotional, social and sexual functions and suggested that nurses in particular are ideally placed to improve the participation of cancer survivors in exercise programmes [48]. With reference to engagement, a recent systematic review identified that the greatest barrier to recruitment and participation in exercise trials in cancer survivorship is transport, distance from the intervention site, time, commitments and health concerns [55]. Such barriers ought to be addressed in future research.

This review identified limited research relating to rehabilitative (*n* = 3), nurse-led (*n* = 2), psychosexual (*n* = 2), family-based (*n* = 1), information support (*n* = 1) and nutritional (*n* = 1) interventions. Further research in these areas is required. It is important to note that promising interventions were highlighted, yet the complexity of supportive care needs for men with a urological cancer makes it difficult to choose a single intervention that addresses all care needs. An added complexity is the significant heterogeneity in intervention effectiveness, with some interventions proving effective at certain timepoints only, and others proving ineffective regardless of the length of follow-up. A future systematic review and meta-analysis would provide more clarity around intervention effectiveness. Moreover, a future review of feasibility and pilot studies would help identify the processes needed for an intervention to be effective and mitigate potential sources of intervention failure. There is also scope for another scoping review focussing exclusively on qualitative and quantitative descriptive research whereby the unmet supportive care needs of men with a urological cancer are scrutinised, particularly that past reviews have focused exclusively either on a narrow set of unmet needs [14] or on a single urological cancer, particularly PCa [4–6].

## Limitations

Studies were significantly heterogeneous, particularly in terms of the categories of interventions (*n* = 10 categories), outcomes measured (*n* = 321 outcomes identified) and instruments used (*n*=112 instruments identified). Also, given that this is a scoping review, the review questions and eligibility criteria were kept broad, and findings from the included studies were synthesised narratively. Feasibility, pilot, qualitative and quantitative descriptive studies were not eligible for inclusion in this review, thus increasing the risk of study selection bias. While the intention was to include studies addressing all urological cancers, 29 of the included 30 studies focussed exclusively on PCa. Therefore, review findings cannot be generalised to men with various urological cancers.

## Conclusion

To the best our knowledge, this is the first scoping review to describe the effect of supportive care interventions for men with any urological cancer. Overall, multicomponent, cognitive-behavioural, telephone, web-based, physical activity/exercise-based and rehabilitative interventions were promising in improving various physical and psychological outcomes. This improvement, however, was often short-lived. There is a need for future studies that are powered, longitudinal, controlled and conducted among men with urological cancers other than PCa. All men in the included studies were receiving treatment for their urological cancer. This highlights the need for future research that addresses the needs of and uncertainty experienced by men undergoing watchful waiting. Structural and access barriers ought to be examined and controlled for in future research as these might affect compliance, particularly for complex multicomponent interventions delivered in person.

## Supplementary information

ESM 1 (DOCX 111 kb)

ESM 2 (DOCX 23 kb)

ESM 3 (DOCX 18 kb)

## Data Availability

All generated or analysed data during this study have been included in this published article and supplementary files.
