# Supplementary material for: Supportive care interventions for men with urological cancers: a scoping review
Source: Support Care Cancer. 2023 Aug 21;31(9):530. doi: 10.1007/s00520-023-07984-0 (PMC10442278; doi:10.1007/s00520-023-07984-0)
Supplement: Supplementary file 1 — (DOCX 111 kb) [file 520_2023_7984_MOESM1_ESM.docx]

**Supplemental File 1.** Full data extraction table (n=30 studies)

| **a) Reference**  **b) Country** | **a) Design**  **b) Setting** | **a) Sample size *(At last point of data collection, reported only for patients)***  **b) Cancer type**  **c) Cancer stage *(Advanced/ not advanced/ mixed/ unclear)***  **d) Treatment received**  **e) Dyad *(Dyadic/ not dyadic)*** | **a) Type of intervention**  **b) Person(s) involved in delivery**  **c) Duration of delivery**  **d) Control group *(Where applicable)*** | **Frequency of outcome measurement** | **Outcome measured** | **Instrument/ unit of measurement** | **Results *(Pre-post between or within groups, using p-values only)*** |
| --- | --- | --- | --- | --- | --- | --- | --- |
| a) Badger et al. 2013  b) USA | a) Longitudinal study  b) Regional cancer centers, regional Veterans  Affairs Heath Care Centers, cancer support groups | a) n=64 PC survivors  b) PC  c) Mixed  d) prostatectomy, radiation and hormone therapy  e) Not dyadic | a) Two telephone interventions: THE and TIP-C  b) one master’s prepared social worker and one master’s prepared nurse  c) 8 weeks  d) N/A | Baseline, 8 weeks, 16 weeks | Prostate specific function on depression | CES-D | THE vs TIP-C: 16 weeks: p=0.05-0.09 |
|  |  |  |  |  | Chemotherapy on depression | CES-D | THE vs TIP-C: 16 weeks: p=0.05-0.09 |
|  |  |  |  |  | Social support on depression | CES-D | THE vs TIP-C: 16 weeks: p≤0.05 (Favouring THE: reduced depression when lower social support from friends) |
|  |  |  |  |  | Cancer knowledge on depression | CES-D | THE vs TIP-C: 16 weeks: p=0.05-0.09 |
|  |  |  |  |  | Symptom distress on negative affect | PANAS | THE vs TIP-C: 16 weeks: p=0.05-0.09 |
|  |  |  |  |  | Symptom management on negative affect | PANAS | THE vs TIP-C: 16 weeks: p≤0.05 (Favouring THE: reduced negative affect when lower ability to manage symptoms) |
|  |  |  |  |  | Prostate specific function on positive affect | PANAS | THE vs TIP-C: 16 weeks: p=0.05-0.09 |
|  |  |  |  |  | Social support on positive affect | PANAS | THE vs TIP-C: 16 weeks: p≤0.05 (Favouring TIP-C: increased positive affect when social support from friends) |
|  |  |  |  |  | Cancer knowledge on positive affect | PANAS | THE vs TIP-C: 16 weeks: p=0.05-0.09 |
| a) Badger et al. 2011  b) USA | a) Longitudinal study  b) Regional cancer centers, Veterans  Administration centers, cancer support groups, oncologists office | a) n=70 PC survivors; n=70 family partners  b) PC  c) Mixed  d) prostatectomy, radiation and hormone therapy  e) dyadic | a) Two telephone interventions: HEAC and TIP-C  b) one master’s prepared social worker and one master’s prepared nurse  c) 8 weeks  d) N/A | Baseline, 8 weeks, 16 weeks | Depression | CES-D | HEAC vs TIP-C:16 weeks: p<0.001 (Favouring HEAC) |
|  |  |  |  |  | Positive affect | PANAS | HEAC vs TIP-C: 8 weeks: p<0.001  HEAC vs TIP-C:16 weeks: Not statistically significant (p Value NR) |
|  |  |  |  |  | Negative affect | PANAS | HEAC vs TIP-C: 16 weeks: p<0.001 (Favouring HEAC) |
|  |  |  |  |  | Perceived stress | PSS | HEAC vs TIP-C: 16 weeks: p<0.001 (Favouring HEAC) |
|  |  |  |  |  | UCLA Prostate cancer Index | UCLA Prostate cancer Index | HEAC vs TIP-C: 16 weeks: Not statistically significant (p Value NR) |
|  |  |  |  |  | Social Support-family | PSS-FA | HEAC vs TIP-C: 16 weeks: Not statistically significant (p Value NR) |
|  |  |  |  |  | Social wellbeing | Social wellbeing scale | HEAC vs TIP-C: 16 weeks: Not statistically significant (p Value NR) |
|  |  |  |  |  | Spiritual wellbeing | Spiritual wellbeing subscale | HEAC vs TIP-C: 16 weeks: p<0.01 (Favouring HEAC) |
|  |  |  |  |  | Fatigue | MFI | HEAC vs TIP-C: 8 weeks: p<0.001  HEAC vs TIP-C: 16 weeks: p<0.001 (Favouring HEAC) |
| a) Beydoun et al. (2014)  b) Australia | a) Prospective study  b) Community-based | a) n=859 (n=255: at home exercise group; n=396: face to face exercise group; n=208: man plan support group)  b) PC  c) Unclear  d) ADT  e) Not dyadic | a) Exercise intervention with three interventional streams including group  (Face-to-Face) exercise sessions, home-based (At Home) exercise or a support programme for those incapable of exercising (Support)  b) (i) At home exercise group= Health coach  (ii) Face to face exercise group=AEP  c) (i) At home exercise group= 6 months  (ii) Face to face exercise group= 10 weeks followed by at home exercise for 6 months  d) N/A | (i) Face to face programme: Baseline, 10 weeks, 6 months  (ii) At home programme: 3 months, 6 months | Weight | Kg | 10 weeks: p=0.466 |
|  |  |  |  |  | BMI | Kg/m | 10 weeks: p=0.544 |
|  |  |  |  |  | Waist circumference | cm | 10 weeks: p≤0.0001 (favouring face to face programme: reduced waist circumference) |
|  |  |  |  |  | Hip circumference | cm | 10 weeks: p=0.015 (favouring face to face programme: reduced hip circumference) |
|  |  |  |  |  | Resting heart rate (Cardiovascular and cardiorespiratory fitness variable) | bpm | 10 weeks: p=0.282 |
|  |  |  |  |  | Completion of 400m walk (Cardiovascular and cardiorespiratory fitness variable) | Sec | 10 weeks: p≤0.0001 (favouring face to face programme: reduced mean time) |
|  |  |  |  |  | DBP | mmHg | 10 weeks: p≤0.0001 (favouring face to face programme: reduced DBP) |
|  |  |  |  |  | SBP | mmHg | 10 weeks: p=0.0044 (favouring face to face programme: reduced SBP) |
|  |  |  |  |  | Leg press, bench press, seated row, knee push ups, toe push ups, abdominal strength (all strength variables) | Kg, reps, sec | 10 weeks: p≤0.0001 (all strength variables) (favouring face to face programme) |
| a) Bouchard et al. (2019)  b) USA | a) RCT  b) Two medical centres | a) n=154 (Intervention=77; control=77)  b) PC  c) Advanced (stages III–IV)  d) ADT  e) Not dyadic | a) Psychosocial intervention (CBSM)  b) Group facilitators (masters level therapists)  c) 10 weeks  d) HP control | Baseline, 6 months, 12 months | Anxiety (total sample) | MAX-PC | CBSM vs HP: p =0.034  CBSM vs HP: 6 months: p=0.011  CBSM vs HP: 12 months: p>0.05 |
|  |  |  |  |  | Anxiety (CBSM vs HP) | MAX-PC | CBSM:  6 months: p<0.001  12 months: p=0.011  HP:  6 months: p>0.05  12 months: p>0.05  (Favouring intervention: reduced anxiety) |
| a) Bourke et al. (2014)  b) UK | a) RCT  b) Outpatient clinics | a) n=68 (n=35 intervention group and n=33 control group)  b) PC  c) Advanced  d) ADT  e) Not dyadic | a) Combined tapered  exercise and dietary advice with integrated behaviour change support  b) Exercise physiologist  c) 12 weeks  d) Usual care | Baseline, 12 weeks post-test, 6 months post-test | Disease-specific QoL | FACT-P | 12 weeks: p=0.001  6 months: p=0.27 |
|  |  |  |  |  | Patient reported fatigue | FACT-F | 12 weeks: p<0.001  6 months: p<0.007 (Favouring intervention) |
|  |  |  |  |  | Total exercise behaviour | Godin Leisure Score Index | 12 weeks: p<0.001  6 months: p=0.038 (Favouring intervention) |
|  |  |  |  |  | Aerobic exercise  tolerance | Symptom-limited graded exercise test | 12 weeks: p<0.001  6 months: p<0.001 (Favouring intervention) |
|  |  |  |  |  | Systolic blood pressure | British Hypertension Society Guidance | 12 weeks: p=0.2  6 months: p=0.89 |
|  |  |  |  |  | BMI | Height (wall-mounted stadiometer) and weight (Weylux beam balance scales) | 12 weeks: p=0.71  6 months: p=0.15 |
|  |  |  |  |  | Dietary behaviour | 3-d diet diaries analysed using NetWisp v.3.0 | 12 weeks:   - Total fat: p=0.039 - Saturated fat: p=0.013 - Monosaturated fat: p=0.047   (Favouring intervention: reduced intake of fats) |
|  |  |  |  |  | Serum PSA | Immunoenzymatic assay | 12 weeks post-test: p=0.41 |
| a) Carmack Taylor et al. (2006)  b) USA | a) RCT  b) Community centres | a) n=113 (lifestyle program n=35; educational support n=44; standard care n=31)  b) PC  c) Unclear  d) ADT  e) Not dyadic | a) Lifestyle physical activity intervention  b) (i) Lifestyle program=physical therapists (ii) educational support program= interventionists  c) 6 months  d) Usual care | Baseline, 6 months, 12 months | Physical composite, mental composite, social functioning, general health, physical functioning, physical role, emotional role, and mood | SF-36 | 6 months: No statistically significant differences (p-value NR)  12 months: No statistically significant differences (p-value NR) |
|  |  |  |  |  | Bodily Pain | BPI | 6 months: No statistically significant differences (p-value NR)  12 months: No statistically significant differences (p-value NR) |
|  |  |  |  |  | Endurance | Six-minute walk test | 6 months: No statistically significant differences (p-value NR)  12 months: No statistically significant differences (p-value NR) |
|  |  |  |  |  | Body composition/ BMI | cm | 6 months: No statistically significant differences (p-value NR)  12 months: No statistically significant differences (p-value NR) |
|  |  |  |  |  | Social support | ISEL | 6 months: No statistically significant differences (p-value NR)  12 months: No statistically significant differences (p-value NR) |
|  |  |  |  |  | Physical activity | days/week active for >30 min | 6 months: No statistically significant differences (p-value NR)  12 months: No statistically significant differences (p-value NR) |
|  |  |  |  |  | Energy expenditure | kcal/kg/day | 6 months: No statistically significant differences (p-value NR)  12 months: No statistically significant differences (p-value NR) |
|  |  |  |  |  | Processes of change for physical activity | Processes of Change for Physical Activity Questionnaire | 6 months: p=0.02  12 months: p=0.63 |
|  |  |  |  |  | Decisional balance for physical activity | Decisional Balance for Physical Activity Questionnaire | 6 months: p=0.42  12 months: p=0.81 |
|  |  |  |  |  | Self-efficacy for physical activity | Physical activity self-efficacy questionnaire | 6 months: p=0.09  12 months: p=0.2 |
| a) Chambers et al. (2019)  b) Australia | a) RCT  b) Private clinics and public hospitals | a) n=107 (n=41 nurse, n=38 peer, n=28 usual care)  b) PC  c) Not advanced  d) Scheduled for or have undergone radical prostatectomy in past 12 months  e) Dyadic | a) Two psychosexual intervention arms including a peer support group and nurse counselling group  b) Nurse counsellors or peer-support volunteers  c) 5 years  d) Usual care | Baseline, 3 months, 6 months, 12 months, 2 years, 3 years, 4 years, 5 years | Sexual self confidence | IIEF | Peer group vs control (favouring control):  5 years: p=0.043 |
|  |  |  |  |  | Sexual function and satisfaction | IIEF | No statistically significant differences (p-value NR) |
|  |  |  |  |  | Masculine self esteem | The Masculine Self‐Esteem Scale | Nurse group vs control (favouring nurse group intervention):  2 years: p=0.052  5 years: p=0.045 |
|  |  |  |  |  | Sexual supportive care needs | Supportive Care Needs Survey | Peer group vs control (control group had less sexual supportive care needs  than peer group and nurse group)  3 years: p=0.001 (peer group)  3 years: p=0.01 (nurse group) |
|  |  |  |  |  | Use of treatments for erectile dysfunction | Schover scale | No statistically significant differences (p-value NR) |
|  |  |  |  |  | Overall use of treatments for sexual problems | Unclear | Peer group vs control (peer group used more treatments than control)  2 years: p=0.060  3 years: p=0.040  4 years: p=0.002  5 years: p=0.005  Nurse group vs control (nurse group used more treatments than control)  2 years: p=0.001  3 years: p=0.014  4 years: p=0.004  5 years: p=0.007 |
| a) Cohen et al. (2011)  b) USA | a) RCT  b) Medical centre | a) n=127 (stress management: n=38; supportive attention: n=45; standard care: n=44)  b) PC  c) Not advanced  d) Undergoing radical prostatectomy  e) Not dyadic | a) Two intervention arms including stress management (discussion of concerns about surgery,  diaphragmatic breathing, guided imagery, imaginal exposure to the day of surgery, adaptive coping skills) and supportive attention (discussion of concerns about upcoming surgery and medical interview)  b) Research assistant and psychologist  c) NR  d) Usual care | Baseline, 1 week before surgery, morning of surgery, 48 hours after surgery | Mood disturbance | POMS | Stress management vs control: 1 week before surgery: p=0.006 (favouring stress management intervention: lower mood disturbance)  No other group comparisons reached  significance. |
| a) Dieperink et al. (2013)  b) Denmark | a) RCT  b) Outpatient hospital setting (Department of Oncology and the Department of Rehabilitation) | a) n=161 (Intervention: n-79; control: n=82)  b) PC  c) Mixed  d) ADT and radiotherapy  e) Not dyadic | a) Rehabilitation intervention involving nursing counselling sessions and instructive sessions with a physical therapist  b) Nurse and physical therapists  c) 20 weeks  d) Usual care | Before radiotherapy, 4 weeks after radiotherapy (baseline), 20 weeks, 24 weeks | Physical QoL | SF-12 | 24 weeks: p=0.002 (favouring intervention) |
|  |  |  |  |  | Mental QoL | SF-12 | 24 weeks: p=0.549 |
|  |  |  |  |  | Urinary (sum score) | EPIC | 24 weeks: p=0.023 (favouring intervention) |
|  |  |  |  |  | Urinary incontinence symptoms | EPIC | 24 weeks: p=0.242 |
|  |  |  |  |  | Urinary irritative symptoms | EPIC | 24 weeks: p=0.011 (favouring intervention) |
|  |  |  |  |  | Bowel symptoms | EPIC | 24 weeks: p=0.224 |
|  |  |  |  |  | Sexual symptoms | EPIC | 24 weeks: p=0.117 |
|  |  |  |  |  | Hormonal symptoms | EPIC | 24 weeks: p=0.018 (favouring intervention) |
|  |  |  |  |  | Digital evaluation | Modified Oxford Scale | No statistically significant differences (p-value NR) |
|  |  |  |  |  | Static strength no. of seconds to hold one contraction | Modified Oxford Scale | No statistically significant differences (p-value NR) |
|  |  |  |  |  | Dynamic strength no. of contractions during 60s | Modified Oxford Scale | No statistically significant differences (p-value NR) |
| a) Dieperink et al. (2017)  b) Denmark | a) RCT  b) Single-centre oncology unit | a) n=161 (intervention: n=79; control: n=82)  b) PC  c) Mixed  d) ADT and radiotherapy  e) Not dyadic | a) Rehabilitation intervention involving nursing counselling sessions and instructive sessions with a physical therapist  b) Nurse and physical therapists  c) 20 weeks  d) Usual care | Baseline, 6 months, 3 years | Fighting spirit | Mini-MAC | 6 months: p=0.025  3 years: p=0.585 |
|  |  |  |  |  | Fatalism | Mini-MAC | 6 months: p=0.991  3 years: p=0.841 |
|  |  |  |  |  | Cognitive avoidance | Mini-MAC | 6 months: p=0.853  3 years: p=0.044 (favouring intervention: lower cognitive avoidance) |
|  |  |  |  |  | Anxious preoccupation | Mini-MAC | 6 months: p=0.841  3 years: p=0.733 |
|  |  |  |  |  | Helplessness/ Hopelessness | Mini-MAC | 6 months: p=0.076  3 years: p=0.763 |
| a) Faithfull et al. (2022)  b) UK | a) RCT  b) Hospital (radiotherapy centre) | a) n=56 (n=26 intervention group and n=30 control group) b) PC  c) Mixed d) EBRT with neo-adjuvant or adjuvant ADT or low-dose rate radiotherapy  e) Not dyadic | a) SMaRT group intervention based on the rehabilitative pathway (Group support, education and pelvic floor muscle exercises)  b) CNS  c) 10 weeks  d) Control group received usual care | Baseline, 3 months post-baseline, 6 months post-baseline | Urinary symptoms | IPSS | 3 months: p=0.066  6 months: p=0.054 |
|  |  |  |  |  | Voiding symptoms | ICSmaleVS | 3 months: p=0.017  6 months: p=0.521 |
|  |  |  |  |  | Incontinence symptoms | ICSmaleIS | 3 months: p=0.029  6 months: p=0.073 |
|  |  |  |  |  | Symptom-related QoL | EORTC QLQ-PR25 urinary domain | 3 months: p=0.506  6 months: p=0.245 |
|  |  |  |  |  | Emotional distress | EORTC QLQ-30 emotional functioning domain | 3 months: p=0.147  6 months: p=0.902 |
|  |  |  |  |  | Self-efficacy for performing daily activities | SESCI | 3 months: p=0.901  6 months: p=0.324 |
|  |  |  |  |  | Self-efficacy for coping with symptoms | SESCI | 3 months: p=0.274  6 months: p=0.192 |
|  |  |  |  |  | Self-efficacy for managing symptoms | SESCI | 3 months: p=0.017  6 months: p=0.133 |
| a) Forslund et al. (2019)  b) Sweden | a) RCT  b) Hospital | a) n=180 (intervention: n=92; control: n=88)  b) PC  c) Mixed  d) radiotherapy (IMRT) + brachytherapy boost  e) Not dyadic | a) Nutrition intervention  b) Research dietician  c) 8 weeks  d) SC | Baseline, 4 weeks, 8 weeks, 2 months, 7 months, 12 months, 18 months, 24 months | Diarrhoea | EORTC QLQ-C30 | No statistically significant differences (p value NR) |
|  |  |  |  |  | Constipation | EORTC QLQ-C30 | No statistically significant differences (p value NR) |
|  |  |  |  |  | Bloated abdomen | QLQ-PR25 | 7 months: p=0.029  12 months: p=0.029  18 months: p=0.029  24 months: p=0.029 (Favouring control: less bloated abdomen) |
|  |  |  |  |  | Patient Perceived Bother (Eight Bowel Symptoms) | GISEQ | Flatulence:  4 weeks: p=0.014  8 weeks: p=0.014  2 months: p=0.014  (Favouring intervention: less flatulence)  Blood in stools:  4 weeks: p=0.047  8 weeks: p=0.047  2 months: p=0.047  (Favouring intervention: less blood in stools) |
|  |  |  |  |  | Loss of appetite | EORTC QLQ-C30 | 4 weeks: p=0.018  8 weeks: p=0.018  2 months: p=0.018 (favouring intervention: increased loss of appetite) |
|  |  |  |  |  | HRQoL | EORTC QLQ-C30 | No statistically significant differences (p-value NR) |
|  |  |  |  |  | Dietary Adherence | FFQ | No statistically significant differences (p-value NR) |
| a) Giesler et al. (2005)  b) USA | a) RCT  b) Medical and cancer centres | a) n=99 (patients and their partners)  b) PC  c) Not advanced  d) external beam radiation, or brachytherapy  e) Dyadic | a) Nurse Led cancer care intervention  b) Oncology nurse  c) 6 months  d) SC | 4 months, 7 months, 12 months | Sexual function | PCQoL | 4 months: p=0.05  7 months: p=0.10  12 months: p=0.10 |
|  |  |  |  |  | Sexual limitation | PCQoL | 4 months: p=0.09  7 months p=0.05  12 months: p=0.02 (favouring intervention) |
|  |  |  |  |  | Sexual bother | PCQoL | 4 months: p=0.63  7 months: p=0.25  12 months: p=0.34 |
|  |  |  |  |  | Urinary function | PCQoL | 4 months: p=0.14  7 months: p=0.44  12 months: p=0.49 |
|  |  |  |  |  | Urinary limitation | PCQoL | 4 months: p=0.53  7 months: p=0.34  12 months: p=0.28 |
|  |  |  |  |  | Urinary bother | PCQoL | 4 months: p=0.75  7 months: p=0.19  12 months: p=0.53 |
|  |  |  |  |  | Bowel function | PCQoL | 4 months: p=0.26  7 months: p=0.25  12 months: p=0.34 |
|  |  |  |  |  | Bowel limitation | PCQoL | 4 months: p=0.80  7 months: p=0.76  12 months: p=0.86 |
|  |  |  |  |  | Bowel bother | PCQoL | 4 months: p=0.18  7 months: p=0.58  12 months: p=0.53 |
|  |  |  |  |  | Cancer worry | PCQoL | 4 months: p=0.19  7 months: p=0.38  12 months: p=0.03 (favouring intervention: reduced cancer worry) |
|  |  |  |  |  | Depression and urinary bother | CES-D | Patients below the CES-D median score:  4 months: p=0.015  7 months: p=0.007  12 months: p=0.159 |
|  |  |  |  |  | Depression and urinary bother | CES-D | Patients above the CES-D median score:  4 months: p=0.069  7 months: p=0.361  12 months: p=0.058 |
|  |  |  |  |  | Depression and emotional role function | CES-D | 12 months: p=0.784 (patients below the CES-D median score)  12 months: p=0.098 (patients above the CES-D median score) |
|  |  |  |  |  | Depression and physical role function | CES-D | 12 months: p=0.948 (patients below the CES-D median score)  12 months: p=0.014 (patients above the CES-D median score)  (Favouring intervention: depression= greater gains in physical role functioning) |
|  |  |  |  |  | Physical functioning | SF-36 | 4 months: p=0.55  7 months: p=0.99  12 months: p=0.83 |
|  |  |  |  |  | Physical role | SF-36 | 4 months: p=0.15  7 months: p=0.17  12 months: p=0.014 (favouring intervention) |
|  |  |  |  |  | Bodily pain | SF-36 | 4 months: p=0.27  7 months: p=0.27  12 months: p=0.30 |
|  |  |  |  |  | Vitality | SF-36 | 4 months: p=0.84  7 months: p=0.44  12 months: p=0.46 |
|  |  |  |  |  | Dyadic cohesion | DAS | 4 months: p=0.93  7 months: p=0.43  12 months: p=0.75 |
|  |  |  |  |  | Dyadic satisfaction | DAS | 4 months: p=0.25  7 months: p=0.31  12 months: p=0.10 |
|  |  |  |  |  | Emotional role functioning | SF-36 | 4 months: p=0.33  7 months: p=0.9  12 months: p=0.19 |
|  |  |  |  |  | General health perceptions | SF-36 | 4 months: p=0.91  7 months: p=0.85  12 months: p=0.70 |
|  |  |  |  |  | Social functioning | SF-36 | 4 months: p=0.64  7 months: p=0.99  12 months: p=0.35 |
| a) Goode et al. (2022)  b) USA | a) RCT  b) Urology clinics | a) n=245 (intervention: n=123; control: n=122)  b) PC  c) Unclear  d) NR  e) Not dyadic | a) Perioperative behavioral program (education, pelvic floor muscle training, progressive exercises, bladder control techniques)  b) Delivered via mhealth  c) 1-4 weeks preoperatively and 8 weeks postoperatively  d) general prostate cancer education | Baseline, 3 months, 6 months, 9 months, 12 months | Time to achieve continence after surgery | ICIQ SF | Following surgery: p=0.8  6 months: p=0.7 |
|  |  |  |  |  | HrQoL | EPIC-UI | No statistically significant differences (p-value NR) |
|  |  |  |  |  | Pad use | EPIC  IIQ-SF  IPSS QoL | 3 months: p>0.9  6 months: p=0.6  9 months: p=0.8  12 months: p=0.7 |
|  |  |  |  |  | Impact of incontinence on daily activities | ICIQ | 3 months: p=0.2  6 months: p=0.6  9 months: p=0.9  12 months: p=0.8 |
|  |  |  |  |  | Impact of incontinence on QoL | IPSS QoL | 3 months: p=0.9  6 months: p=0.6  9 months: p=0.5  12 months: p=0.8 |
|  |  |  |  |  | Perceived symptom improvement | ICIQ | 3 months: p=0.7  6 months: p=0.7  9 months: p=0.6  12 months: p=0.7 |
|  |  |  |  |  | Patient satisfaction | ICIQ | 3 months: p=0.07  6 months: p=0.04  9 months: p=0.2  12 months: p=0.5 |
|  |  |  |  |  | Resumption of normal activities | ICIQ | 3 months: p=0.4  6 months: p=0.4  9 months: p=0.8  12 months: p=0.8 |
|  |  |  |  |  | Return to work | ICIQ | 3 months: p=0.8  6 months: p=0.7  9 months: p≥0.9  12 months: p=0.8 |
|  |  |  |  |  | Incontinence severity | ICIQ | 3 months: p≥0.9  6 months: p=0.4  9 months: p=0.7  12 months: p=0.17 |
| a) Leahy et al.  (2012)  b) Australia | a) Two cohort comparative study (non-randomised)  b) Outpatient clinics | a) n=169 (intervention: n=86; control: n=83)  b) PC  c) Mixed  d) external beam radiotherapy  e) not dyadic | a) nurse led telephone consultations  b) CNC  c) 6 months  d) N/A | 6 months, 12 months | Patient satisfaction | Satisfaction with Consultation Scale | Low/int risk: p=0.051  High risk: p=0.440 |
|  |  |  |  |  | Distress | Distress Thermometer | Low/int risk: p=1.000  High risk: p=0.831 |
|  |  |  |  |  | Bowel dysfunction | EPIC | No statistically significant differences (p-value NR) |
|  |  |  |  |  | Sexual dysfunction | EPIC | No statistically significant differences (p-value NR) |
|  |  |  |  |  | Hormonal | EPIC | No statistically significant differences (p-value NR) |
|  |  |  |  |  | Urinary irritation | EPIC | No statistically significant differences (p-value NR) |
|  |  |  |  |  | Urinary Incontinence | EPIC | No statistically significant differences (p-value NR) |
| a) Mardani et al. (2021)  b) Iran | a) RCT  b) Park in urban area (community setting) | a) n=71 (n=35 in the intervention group and n=36 in the control group)  b) Prostate cancer  c) not advanced  d) Radiotherapy, hormone therapy and surgery  e) not dyadic | a) Exercise programme including aerobic, resistant, flexible and pelvic floor muscle exercises  b study researcher  c) 12 weeks  d) Control group received usual care | Baseline and after the intervention | Physical function | QLQ-C30 | Baseline p=0.18  Baseline and after the programme p<0.001  After the programme p<0.001  (Favouring intervention) |
|  |  |  |  |  | Role function | QLQ-C30 | Baseline: p=0.72  Baseline and after the programme: p<0.001  After the programme: p=0.002  (Favouring intervention) |
|  |  |  |  |  | Emotional function | QLQ-C30 | Baseline: p=0.42  Baseline and after the programme: p<0.001  After the programme: p=0.35 |
|  |  |  |  |  | Cognitive function | QLQ-C30 | Baseline: p=0.04  Baseline and after the programme: p=0.07  After the programme: p=0.94 |
|  |  |  |  |  | Social function | QLQ-C30 | Baseline: p=0.87  Baseline and after the programme: p<0.001  After the programme: p=0.13 |
|  |  |  |  |  | Global health status and QoL | QLQ-C30 | Baseline: p=0.39  Baseline and after the programme: p=0.79  After the programme: p=0.41 |
|  |  |  |  |  | Fatigue | QLQ-C30 | Baseline: p=0.35  Baseline and after the programme: p<0.001  After the programme: p<0.001 (favouring intervention: reduced fatigue) |
|  |  |  |  |  | Nausea and vomiting | QLQ-C30 | Baseline: p=0.93  Baseline and after the programme: p=0.23  After the programme: p=0.19 |
|  |  |  |  |  | Pain | QLQ-C30 | Baseline: p=0.002  Baseline and after the programme: p=0.36  After the programme: p=0.36 |
|  |  |  |  |  | Dyspnoea | QLQ-C30 | Baseline: p=0.24  Baseline and after the programme: p=0.08  After the programme: p=0.76 |
|  |  |  |  |  | Insomnia | QLQ-C30 | Baseline: p=0.36  Baseline and after the programme: p<0.001  After the programme: p=0.26 |
|  |  |  |  |  | Appetite loss | QLQ-C30 | Baseline: p=0.52  Baseline and after the programme: p=0.79  After the programme: p=0.58 |
|  |  |  |  |  | Constipation | QLQ-C30 | Baseline: p=0.35  Baseline and after the programme: p=0.03  After the programme: p=0.96 |
|  |  |  |  |  | Diarrhoea | QLQ-C30 | Baseline: p=0.002  Baseline and after the programme: p=0.005  After the programme: p=0.37 |
|  |  |  |  |  | Financial difficulties | QLQ-C30 | Baseline: p=0.26  Baseline and after the programme: p=0.15  After the programme: p=0.46 |
|  |  |  |  |  | Sexual activity | QLQ-PR25 | Baseline: p=0.10  Baseline and after the programme: p=0.16  After the programme: p=0.001 (favouring intervention) |
|  |  |  |  |  | Sexual function | QLQ-PR25 | Baseline: p=0.28  Baseline and after the programme: p=0.01  After the programme: p=0.67 |
|  |  |  |  |  | Urinary symptoms | QLQ-PR25 | Baseline: p=0.22  Baseline and after the programme: p<0.001  After the programme: p=0.07 (favouring intervention) |
|  |  |  |  |  | Bowel symptoms | QLQ-PR25 | Baseline: p=0.31  Baseline and after the programme: p<0.001  After the programme: p=0.29 |
|  |  |  |  |  | Incontinence aid | QLQ-PR25 | Baseline: p=0.56  Baseline and after the programme: p=0.31  After the programme: p=0.32 |
|  |  |  |  |  | Hormonal treatment-related symptoms | QLQ-PR25 | Baseline: p=0.051  Baseline and after the programme: p=0.001  After the programme: p=0.77 |
| a) Mareschal et al. (2017)  b) Switzerland | a) Longitudinal multidisciplinary interventional study  b) Hospital | a) n=29  b) PC  c) Mixed  d) Radiation therapy and ADT  e) Not dyadic | a) Nutritional, physical and psychological coaching b) CNS, dietician, physiotherapist and psychologist  c) 24 months  d) N/A | Start of AD, 3, 6, 9, 12, 18 and 24 months and 12 months post study follow up | Urinary dysfunction | PC QoL scale | 12 months post study: p=0.071 |
|  |  |  |  |  | Sexual intimacy | PC QoL scale | 12 months post study: p=0.134 |
|  |  |  |  |  | Sexual confidence | PC QoL scale | 12 months post study: p=0.498 |
|  |  |  |  |  | Masculine self-esteem | PC QoL scale | 12 months post study: p=0.342 |
|  |  |  |  |  | Marital affection | PC QoL scale | 12 months post study: p=0.065 |
|  |  |  |  |  | PSA concern | PC QoL scale | 12 months post study: p=0.277 |
|  |  |  |  |  | Health worries | PC QoL scale | 12 months post study: p= 0.028 (favouring multidisciplinary program: less health worries) |
|  |  |  |  |  | Body mass index | Weight divided by height | 12 months post study: p=0.315 |
|  |  |  |  |  | Fat mass index | Bioelectrical impedance analyzer | 12 months post study: p=0.277 |
|  |  |  |  |  | Fat-free mass index | Geneva BIA formula | 12 months post study: p=0.412 |
|  |  |  |  |  | Cancer control | PC QoL scale | 12 months post study: p=0.046 (favouring multidisciplinary program: increased cancer control) |
|  |  |  |  |  | Future outlook | PC QoL scale | 12 months post study: p=0.002 (reduced future outlook) |
|  |  |  |  |  | Hand grip strength | Dynamometer | 12 months post study: p=0.055 |
|  |  |  |  |  | Walking endurance | 6MWT | 12 months post study: p=0.116 |
|  |  |  |  |  | Perceived Exertion | Borg Scale | 12 months post study: p=0.212 |
|  |  |  |  |  | Level of mobility | TUG | 12 months post study: p=0.416 |
|  |  |  |  |  | Decision regrets | MMSE | 12 months post study: p=0.936 |
|  |  |  |  |  | Cognition | MMSE | 12 months post study: p=0.270 |
|  |  |  |  |  | Anxiety | HAD-A | 12 months post study: p=0.466 |
| a) Moynihan et al. (1998) *  b) UK | a) RCT  b) Testicular Tumor Unit of the Royal Marsden Hospital | a) n=141 (n=33 in the intervention group, n=35 in the control group, and n=73 in the non-participant group)  b) Testicular cancer  c) Not advanced  d) Radiation therapy and chemotherapy  e) Not dyadic | a) Adjuvant psychological therapy  b) Mental health nurse  c) 8 weeks  d) Control group received care as usual | Baseline, 2 months, 4 months and 12 months post-intervention | Anxiety | HAD-A | Baseline: p=0.039  12 months: No statistically significant differences (p-value NR) |
|  |  |  |  |  | Depression | HAD-A | Baseline: p=0.026  12 months: No statistically significant differences (p-value NR) |
|  |  |  |  |  | Fighting spirit | Mental adjustment to cancer scale | Baseline: p=0.34  12 months: No statistically significant differences (p-value NR) |
|  |  |  |  |  | Helplessness | Mental adjustment to cancer scale | Baseline: p=0.34  12 months: No statistically significant differences (p-value NR) |
|  |  |  |  |  | Anxious preoccupation | Mental adjustment to cancer scale | Baseline: p=0.011  12 months: No statistically significant differences (p-value NR) |
|  |  |  |  |  | Fatalism | Mental adjustment to cancer scale | Baseline: p=0.060  12 months: No statistically significant differences (p-value NR) |
|  |  |  |  |  | Denial | Mental adjustment to cancer scale | Baseline: p=0.002  12 months: No statistically significant differences (p-value NR) |
|  |  |  |  |  | Psychological symptoms | Rotterdam symptom checklist | Baseline p<0.001  12 months: No statistically significant differences (p-value NR) |
|  |  |  |  |  | Physical symptoms | Rotterdam symptom checklist | Baseline: p<0.001  12 months: p<0.01 (favouring intervention) |
|  |  |  |  |  | Emotional concealment | Brannon masculinity scale | Baseline: p=0.036  12 months: No statistically significant differences (p-value NR) |
|  |  |  |  |  | Sexual adjustment | Rieker sexual adjustment scale | Baseline: p=0.028  12 months: No statistically significant differences (p-value NR) |
|  |  |  |  |  | Healthcare orientation | Psychological adjustment to illness scale | Baseline: p=0.21  12 months: No statistically significant differences (p-value NR) |
|  |  |  |  |  | Vocational environment | Psychological adjustment to illness scale | Baseline: p=0.20  12 months: p<0.05 (favouring intervention) |
|  |  |  |  |  | Domestic | Psychological adjustment to illness scale | Baseline: p=0.016  12 months: No statistically significant differences (p-value NR) |
|  |  |  |  |  | Sexual relationship | Psychological adjustment to illness scale | Baseline p=0.95  12 months: No statistically significant differences (p-value NR) |
|  |  |  |  |  | Extended family | Psychological adjustment to illness scale | Baseline: p=0.46  12 months: No statistically significant differences (p-value NR) |
|  |  |  |  |  | Social environment | Psychological adjustment to illness scale | Baseline: p=0.41  12 months: No statistically significant differences (p-value NR) |
|  |  |  |  |  | Psychological distress | Psychological adjustment to illness scale | Baseline: p=0.71  12 months: No statistically significant differences (p-value NR) |
| a) Northouse et al. (2007)  b) USA | a) RCT  b) Patient’s home | a) n=218 (n=104 in the intervention group and n=114 in the control group)  b) PC  c) not advanced  d) N/A  e) Dyadic | a) Family-based intervention (FOCUS Program)  b) Intervention nurses  c) 5 sessions within first 4 months  d) Control group received standard clinic care | a) Baseline, 4 months, 8 months and 12 months post-baseline | Quality of life - physical | MOS SF-12 | 4 months: p=0.96  8 months: p=0.80  12 months: p=0.88 |
|  |  |  |  |  | Quality of life - mental | MOS SF-12 | 4 months: p=0.53  8 months: p=0.69  12 months: p=0.96 |
|  |  |  |  |  | Overall Quality of life | FACT-G | 4 months: p=0.10  8 months: p=0.89  12 months: p=0.77 |
|  |  |  |  |  | Appraisal of illness | 27-item Appraisal of Illness | 4 months: p=0.22  8 months: p=0.81  12 months: p=0.74 |
|  |  |  |  |  | Uncertainty | 28-item Mishel Uncertainty in Illness Scale | 4 months: p=0.03  8 months: p=0.57  12 months: p=0.97 |
|  |  |  |  |  | Hopelessness | 20-item Beck Hopelessness Scale | 4 months: p=0.07  8 months: p=0.88  12 months: p=0.67 |
|  |  |  |  |  | Self-efficacy | 17-item Lewis Cancer Self-efficacy Scale | 4 months: p=0.98  8 months: p=0.68  12 months: p=0.57 |
|  |  |  |  |  | Communication | 32-item Lewis Mutuality and Interpersonal Sensitivity Scale | 4 months: p=0.03  8 months: p=0.34  12 months: p=0.85 |
|  |  |  |  |  | Active coping | 28-item Brief Coping Orientations to Problems Experienced scale | 4 months: p=0.69  8 months: p=0.34  12 months: p=0.14 |
|  |  |  |  |  | Avoidant coping | 28-item Brief Coping Orientations to Problems Experienced scale | 4 months: p=0.53  8 months: p=0.14  12 months: p=0.15 |
|  |  |  |  |  | Symptom distress | 16-item Symptom Scale of the OSQ | 4 months: p=0.60  8 months: p=0.45  12 months: p=0.59 |
|  |  |  |  |  | Urinary symptoms | 50-item EPIC | 4 months: p=0.09  8 months: p=0.32  12 months: p=0.67 |
|  |  |  |  |  | Bowel symptoms | 50-item EPIC | 4 months: p=0.57  8 months: p=0.60  12 months: p=0.44 |
|  |  |  |  |  | Sexual symptoms | 50-item EPIC | 4 months: p=0.72  8 months: p=0.24  12 months: p=0.71 |
|  |  |  |  |  | Hormonal symptoms | 50-item EPIC | 4 months: p=0.95  8 months: p=0.97  12 months: p=0.36 |
| a) Penedo et al. (2020) **  b) USA | a) RCT  b) Online (web-based platform) | a) n=154 (n=77 in the intervention group and n=77 in the control group  b) Prostate cancer  c) Advanced  d) ADT  e) Not dyadic | a) Cognitive-behavioral stress and self-management skills with relaxation skills training  b) Master’s or doctoral-level therapists  c) 10 weeks  d) Control received health promotion conditions | Baseline, 6 months and 12 months post-baseline | PC specific symptoms burden (urinary, bowel, sexual and hormonal) | EPIC | CBSM vs HP: not statistically significant (p-value not reported) |
|  |  |  |  |  | HRQoL | FACT-G | CBSM vs HP: not statistically significant (P-value not reported) |
|  |  |  |  |  | Stress management skills and self-efficacy | MOCS | CBSM vs HP: Baseline: p=0.004 |
|  |  |  |  |  | Cancer related anxiety | MAX-PC | CBSM vs HP: Baseline: p=0.023 |
|  |  |  |  |  | Fear of reoccurrence | MAX-PC | Baseline: p=0.010  After adjusting for age: p=0.006 |
| a) Segrin et al. 2012  b) USA | a) Longitudinal study  b) Regional cancer centers, Veterans  Administration centers, cancer support groups, oncologists office | a) n=70 PC survivors; n=70 family partners  b) PC  C) Mixed  d) prostatectomy, radiation and hormone therapy  e) dyadic | a) Two telephone interventions: THE and TIP-C  b) one master’s prepared social worker and one master’s prepared nurse  c) 8 weeks  d) N/A | Baseline, 8 weeks, 16 weeks | Urinary function on depression | CES-D | p<0.001 |
|  |  |  |  |  | Bowel function on depression | CES-D | p<0.001 |
|  |  |  |  |  | Sexual function on depression | CES-D | p<0.05 |
|  |  |  |  |  | PCI on depression | CES-D | p<0.001 |
|  |  |  |  |  | Urinary function on anxiety | STAI | p<0.001 |
|  |  |  |  |  | Bowel function on anxiety | STAI | p<0.001 |
|  |  |  |  |  | Sexual function on anxiety | STAI | p=0.05-0.08 |
|  |  |  |  |  | PCI on anxiety | STAI | p<0.001 |
|  |  |  |  |  | Urinary function on fatigue | MFI | p<0.01 |
|  |  |  |  |  | Bowel function on fatigue | MFI | p<0.001 |
|  |  |  |  |  | Sexual function on fatigue | MFI | p<0.01 |
|  |  |  |  |  | PCI on fatigue | MFI | p<0.001 |
|  |  |  |  |  | Urinary function on positive affect | PANAS | p<0.05 |
|  |  |  |  |  | Bowel function on positive affect | PANAS | p<0.001 |
|  |  |  |  |  | Sexual function on positive affect | PANAS | No significant differences (P-value NR) |
|  |  |  |  |  | PCI on positive affect | PANAS | p<0.001 |
| a) Skolarus et al. (2019)  b) USA | a) RCT  b) Four Veterans Affairs sites | a) n=556 (n=278 in the intervention group and n=278 in the control group)  b) PC  c) N/A  d) surgery, radiation or ADT  e) not dyadic | a) Behavioral: Interactive Voice Response Symptom Management and Tailored Newsletters  b) Patient (automated phone assessment)  c) 3 months  d) Enhanced usual care | Baseline, 5 and 12 months | Urinary Health – incontinence | EPIC | 5 months p=0.11  12 months p=0.90 |
|  |  |  |  |  | Urinary Health – Irritative/Obstructive | EPIC | 5 months p=0.10  12 months p=0.58 |
|  |  |  |  |  | Bowel Health | EPIC | 5 months p=0.75  12 months p=0.16 |
|  |  |  |  |  | Sexual Health | EPIC | 5 months p=0.19  12 months p=0.17 |
|  |  |  |  |  | General Health | EPIC | 5 months p=0.75  12 months p=0.78 |
|  |  |  |  |  | Confidence in Symptom-Self Management | 5-item scale | 5 months p=0.56  12 months p=0.03 (Favouring intervention) |
|  |  |  |  |  | Cancer Control | Cancer control items scale | 5 months p=0.92  12 months p=0.34 |
|  |  |  |  |  | Cancer Outlook | Cancer outlook items scale | 5 months p=0.80  12 months p=0.09 |
|  |  |  |  |  | Self-efficacy in patient-physician interactions | PEPPI | 5 months p=0.48  12 months p=0.29 |
|  |  |  |  |  | Brief cope | Brief Cope instrument | 5 months p=0.02  12 months p=0.21 |
|  |  |  |  |  | Veteran Quality of Life – Physical Health | VR-12 | 5 months: not statistically significant (P-value not reported)  12 months p=0.007 (Favouring control) |
|  |  |  |  |  | Veteran Quality of Life – Emotional Health | VR-12 | 5 months: no statistically significant differences (P-value not reported)  12 months p=0.11 |
| a) Tagai et al. (2021)  b) USA | a) RCT  b) Mid-Atlantic cancer centers | a) n=210 (n=103 in the intervention group and n=107 in the control group)  b) PC  c) Not advanced  d) Surgery, radiation and hormone therapy  e) Not dyadic | a) PROGRESS programme, a web-based intervention designed to improve adaptive coping among PCa survivors.  b) Patients (web-based intervention)  c) 6 months  d) Control group received the usual care | Baseline 1 month, 3 and 6 months | Urinary Health – Irritative/Obstructive | EPIC | 6 months: p<0.001 (improvements in both intervention and control) |
|  |  |  |  |  | Urinary incontinence | EPIC | 6 months: p<0.001 (improvements in both intervention and control) |
|  |  |  |  |  | Bowel dysfunction | EPIC | No statistically significant differences (P -value NR) |
|  |  |  |  |  | Sexual dysfunction | EPIC | 6 months: p<0.05 (improvements in both intervention and control) |
|  |  |  |  |  | General coping techniques | The 5-item coping subscale | 6 months: p<0.05 (improvements in both intervention and control) |
|  |  |  |  |  | Planning | 3-item planning subscale | No statistically significant differences (p-value NR) |
|  |  |  |  |  | Positive outlook | 3-item positive subscale | 6 months: p<0.001 (decrease in positive coping in both intervention and control) |
|  |  |  |  |  | Interpersonal coping | 7-item interpersonal coping scale | 6 months: p<0.05 (smaller decrease in interpersonal coping in intervention) |
|  |  |  |  |  | Self-efficacy for symptom control | 13-item self-efficacy for symptom control scale | No statistically significant differences (p-value NR) |
|  |  |  |  |  | Self-efficacy for re-entry | 14-item author-constructed 11-point scale | No statistically significant differences (p-value NR) |
|  |  |  |  |  | Practical concerns | 12-item practical concerns scale | 6 months: p<0.001 (increase in practical concerns in both intervention and control)  PROGRESS user’s vs non users: p<0.05 (favouring PROGRESS users- decrease in practical concerns) |
|  |  |  |  |  | Diversion coping | 3-item diversion subscale | 6 months: p<0.01 (favouring intervention) |
|  |  |  |  |  | Medical interactions | 5-item medical interactions scale | No statistically significant differences (p-value NR) |
|  |  |  |  |  | Marital interactions | 5-item marital interactions scale | 6 months: p<0.05 (favouring control) |
| a) Carmack Taylor et al. (2007)  b) USA | a) RCT  b) Hospital | a) n=113  b) Prostate cancer  c) Unclear  d) Hormone therapy  e) Not dyadic | a) Active for Life program based on cognitive behavioural skills and physical activity  b) Prostate cancer expert speaker  c) 6 months  d) Control group received standard care | a) Baseline, 6 months and 12 months post-intervention | Role physical | SF-36 | Both intervention groups combined vs control 6 months: p=0.05  Both intervention groups combined vs control 12 months: p=0.71 |
|  |  |  |  |  | Bodily pain | SF-36 | Both intervention groups combined vs control 6 months: p=0.07  Education support program vs control: 12 months: p=0.04 (favouring intervention) |
|  |  |  |  |  | Vitality | SF-36 | Both intervention groups combined vs control 6 months: p=0.20  Both intervention groups combined vs control 12 months: p=0.54 |
|  |  |  |  |  | Pain | BPI | Both intervention groups combined vs control 6 months: p=0.002  Both intervention groups combined vs control 12 months: p=0.22 |
|  |  |  |  |  | Six-minute walk | Six-minute walk test | Both intervention groups combined vs control 6 months: p=0.24  Both intervention groups combined vs control 12 months: p=0.55 |
|  |  |  |  |  | Anxiety | STAI | Both intervention groups combined vs control 6 months: p=0.03  Both intervention groups combined vs control 12 months: p=0.08 |
|  |  |  |  |  | Depression | CES-D | Both intervention groups combined vs control 6 months: p=0.03  Both intervention groups combined vs control 12 months: p=0.006  (Favouring intervention) |
|  |  |  |  |  | General Health | SF-36 | Both intervention groups combined vs control 6 months: p=0.11  Both intervention groups combined vs control 12 months: p=0.36 |
|  |  |  |  |  | Self-esteem | ISEL | Both intervention groups combined vs control 6 months: p=0.52  Both intervention groups combined vs control 12 months: p=0.61 |
|  |  |  |  |  | Belonging | ISEL | Both intervention groups combined vs control 6 months: p=0.16  Both intervention groups combined vs control 12 months: p=0.64 |
|  |  |  |  |  | Appraisal | ISEL | Both intervention groups combined vs control 6 months: p=0.58  Both intervention groups combined vs control 12 months: p=0.43 |
|  |  |  |  |  | Tangible | ISEL | Both intervention groups combined vs control 6 months: p=0.003  Both intervention groups combined vs control 12 months: p=0.05 (favouring intervention) |
|  |  |  |  |  | Mental health | SF-36 | Both intervention groups combined vs control 6 months: p=0.57  Both intervention groups combined vs control 12 months: p=0.27 |
|  |  |  |  |  | Role emotional | SF-36 | Both intervention groups combined vs control 6 months: p=0.29  Both intervention groups combined vs control 12 months: p=0.77 |
|  |  |  |  |  | Social function | SF-36 | Both intervention groups combined vs control 6 months: p=0.32  Both intervention groups combined vs control 12 months: p=0.83 |
|  |  |  |  |  | Physical functioning | SF-36 | Both intervention groups combined vs control 6 months: p=0.47  Both intervention groups combined vs control 12 months: p=0.69 |
| a) Winters Stone et al. (2016)  b) USA | a) RCT  b) Oregon Health & Science University | a) n=64 (n=32 in the intervention group and n=32 in the control group)  b) PC  c) Unclear  d) Surgery, radiation therapy and ADT  e) Dyadic | a) Exercising together project providing strength training  b) Exercise physiologist  c) twice weekly for 6 months  d) Control group received care as usual | a) Baseline, 3 months, and 6 months post intervention | Body weight | DXA | 6 months: p= 0.37 |
|  |  |  |  |  | Lean mass | DXA | 6 months: p= 0.81 |
|  |  |  |  |  | Fat mass | DXA | 6 months: p= 0.13 |
|  |  |  |  |  | Trunk mass | DXA | 6 months: p= 0.11 |
|  |  |  |  |  | % body fat | N/A | 6 months: p= 0.13 |
|  |  |  |  |  | Bench press | 1-RM protocol | 6 months: p<0.01 (favouring intervention) |
|  |  |  |  |  | Leg press | 1-RM protocol | 6 months: p=0.19 |
|  |  |  |  |  | Chair stand | 1-RM protocol | 6 months: p=0.77 |
|  |  |  |  |  | Gait speed | 1-RM protocol | 6 months: p=0.80 |
|  |  |  |  |  | Physical performance | Physical performance battery | 6 months: p=0.48 |
|  |  |  |  |  | Physical health | SF-36 | 6 months: p=0.99 |
|  |  |  |  |  | Mental health | SF-36 | 6 months: p=0.39 |
|  |  |  |  |  | Physical function | SF-36 | 6 months: p=0.72 |
|  |  |  |  |  | Vitality | SF-36 | 6 months: p=0.98 |
|  |  |  |  |  | Physical activity | CHAMPS questionnaire | 6 months: p<0.01 (favouring intervention) |
| a) Wittman et al. (2022)  b) USA | a) RCT  b) Online/patient’s home | a) n=192 (n=39 patients, n=30 partners in the intervention group and n=66 patients and n=55 partners in the control group) b) Prostate cancer  c) Mixed  d) Surgery, radiation therapy and ADT  e) Dyadic | a) The TrueNTH Sexual Recovery Intervention  b) Patient  c) 6 modules over 7-month period  d) Control group received care as usual | Baseline, 3 months and 6 months after beginning of the treatment | Satisfaction with sex life | PROMIS | Baseline: p=0.3  6 months: p=0.4 |
|  |  |  |  |  | Mean non-intercourse activities | PROMIS | 3 months: p=0.38  6 months: p=0.41 |
|  |  |  |  |  | Sexual interest | PROMIS | 3 months: p=0.15  6 months: p=0.2 |
|  |  |  |  |  | Sexual activity with nonpenetrative activities | PROMIS | 3 months: p=0.073  6 months: p=0.85 |
|  |  |  |  |  | Sexual activity with increase vaginal penetration | PROMIS | 3 months: p=0.008  6 months: p=0.50 |
|  |  |  |  |  | Sexual Function | EPIC-26 | 3 months: p=0.80  6 months: p=0.40 |
| a) Wootten et al. (2015)  b) Australia | a) RCT  b) Online (Forum) | a) n=104 (n=33 in group 1/MRA Only, n=35 in group 2/ MRA + Forum and n=36 in group 3/Forum only)  b) Prostate cancer  c) Not advanced  d) Radiation therapy, hormone therapy  e) Not dyadic | a) Psychological intervention called My Road Ahead  b) Patient  c) 10 weeks  d) N/A | Baseline, 5 weeks, post intervention (week 10 for group 3), 3 months, 6 months | Health worry | DAS-21 | Pre vs post: p=0.08 |
|  |  |  |  |  | Informed decision | DAS-21 | Pre vs post: p=0.035 (favouring MRA only group) |
|  |  |  |  |  | Regret | DAS-21 | Pre vs post: p=0.047 (favouring MRA+ Forum group) |
|  |  |  |  |  | Outlook | DAS-21 | Pre vs post: p=0.02 (favouring MRA only group: increase in outlook) |
|  |  |  |  |  | General Confidence | DAS-21 | Pre vs post: p=0.33 |
|  |  |  |  |  | Psychological distress | DAS-21 | Pre vs post: p=0.02 (favouring MRA+ Forum group: reduction in psychological distress) |
| a) Yang et al. (2021)  b) China | a) RCT  b) Cancer hospital | a) n=95 (n=51 in the intervention group and n=44 in the control group)  b) Prostate cancer  c) Mixed  d) Hormonal therapy and surgery  e) not dyadic | a) Information support program on the self-efficacy  b) Urology nurse  c) 8 weeks  d) Control group received routine care | Baseline and 3 months post-baseline | Total information acquisition | EORTC QLQ-INFO25 | Baseline comparison of groups: p=0.679  3-month comparison of groups: p=0.000  (Favouring intervention)  Change in experimental group before and after intervention: p=0.000 |
|  |  |  |  |  | Disease information acquisition | EORTC QLQ-INFO25 | Baseline comparison of groups: p=0.889  3-month comparison of groups: p=0.034 (favouring intervention)  Change in experimental group before and after intervention: p=0.042 |
|  |  |  |  |  | Medical examination information acquisition | EORTC QLQ-INFO25 | Baseline comparison of groups: p=0.936  3-month comparison of groups: p=0.674  Change in experimental group before and after intervention: p=0.790 |
|  |  |  |  |  | Treatment information acquisition | EORTC QLQ-INFO25 | Baseline comparison of groups: p=0.647  3-month comparison of groups: p=0.009 (favouring intervention)  Change in experimental group before and after intervention: p=0.001 |
|  |  |  |  |  | Other services information | EORTC QLQ-INFO25 | Baseline comparison of groups: p=0.892  3-month comparison of groups: p=0.000 (favouring intervention)  Change in experimental group before and after intervention: p=0.015 |
|  |  |  |  |  | Medical places (information) | EORTC QLQ-INFO25 | Baseline comparison of groups: p=0.337  3-month comparison of groups: p=0.008 (favouring intervention)  Change in experimental group before and after intervention: p=0.826 |
|  |  |  |  |  | Self-care (information) | EORTC QLQ-INFO25 | Baseline comparison of groups: p=0.872  3-month comparison of groups: p=0.024 (favouring intervention)  Change in experimental group before and after intervention: p=0.450 |
|  |  |  |  |  | Written information | EORTC QLQ-INFO25 | Baseline comparison of groups: p=0.255  3-month comparison of groups: p=0.000 (favouring intervention)  Change in experimental group before and after intervention: p=0.000 |
|  |  |  |  |  | Video information | EORTC QLQ-INFO25 | Baseline comparison of groups: p=0.211  3-month comparison of groups: p=0.000 (favouring intervention)  Change in experimental group before and after intervention: p=0.000 |
|  |  |  |  |  | Information Satisfaction | EORTC QLQ-INFO25 | Baseline comparison of groups: p=0.451  3-month comparison of groups: p=0.003 (favouring intervention)  Change in experimental group before and after intervention: p=0.048 |
|  |  |  |  |  | More information | EORTC QLQ-INFO25 | Baseline comparison of groups: p=0.552  3-month comparison of groups: p=0.126  Change in experimental group before and after intervention: p=0.034 |
|  |  |  |  |  | Less information | EORTC QLQ-INFO25 | Baseline comparison of groups: p=1.00  3-month comparison of groups: p=0.131  Change in experimental group before and after intervention: p=0.317 |
|  |  |  |  |  | Information assistance | EORTC QLQ-INFO25 | Baseline comparison of groups: p=0.866  3-month comparison of groups: p=0.011 (favouring intervention)  Change in experimental group before and after intervention: p=0.982 |
|  |  |  |  |  | Total disease knowledge mastery | Self-designed disease knowledge questionnaire | 3-month comparison of groups: p=0.000 (favouring intervention) |
|  |  |  |  |  | PC disease knowledge mastery | Self-designed disease knowledge questionnaire | 3-month comparison of groups: p=0.001 (favouring intervention) |
|  |  |  |  |  | Follow-up review disease knowledge mastery | Self-designed disease knowledge questionnaire | 3-month comparison of groups: p=0.000  (Favouring intervention) |
|  |  |  |  |  | Hormone therapy disease knowledge mastery | Self-designed disease knowledge questionnaire | 3-month comparison of groups: p=0.000 (favouring intervention) |
|  |  |  |  |  | Healthy lifestyle  disease knowledge mastery | Self-designed disease knowledge questionnaire | 3-month comparison of groups: p=0.006 (favouring intervention) |
|  |  |  |  |  | Total self-efficacy | Strategies Used by People to Promote Health | Baseline comparison of groups: p=0.319  3-month comparison of groups: p=0.044 (favouring intervention)  Change in experimental group before and after intervention: p=0.864 |
|  |  |  |  |  | Positive attitude | Strategies Used by People to Promote Health | Baseline comparison of groups: p=0.267  3-month comparison of groups: p=0.047 (favouring intervention)  Change in experimental group before and after intervention: p=0.772 |
|  |  |  |  |  | Self-decompression | Strategies Used by People to Promote Health | Baseline comparison of groups: p=0.757  3-month comparison of groups: p=0.107  Change in experimental group before and after intervention: p=0.097 |
|  |  |  |  |  | Self-decision making | Strategies Used by People to Promote Health | Baseline comparison of groups: p=0.248  3-month comparison of groups: p=0.035 (favouring intervention)  Change in experimental group before and after intervention: p=0.642 |
|  |  |  |  |  | Total healthy behavior adherence | Self-designed healthy behavior adherence questionnaire | 3-month comparison of groups: p=0.026 (favouring intervention) |
|  |  |  |  |  | Bad habits healthy behavior adherence | Self-designed healthy behavior adherence questionnaire | 3-month comparison of groups: p=0.019 (favouring intervention) |
|  |  |  |  |  | Diet healthy behavior adherence | Self-designed healthy behavior adherence questionnaire | 3-month comparison of groups: p=0.038 (favouring intervention) |
|  |  |  |  |  | Exercise healthy behavior adherence | Self-designed healthy behavior adherence questionnaire | 3-month comparison of groups: p=0.026 (favouring intervention) |
|  |  |  |  |  | Medication healthy behavior adherence | Self-designed healthy behavior adherence questionnaire | 3-month comparison of groups: p=0.533 |
|  |  |  |  |  | Follow-up healthy behavior adherence | Self-designed healthy behavior adherence questionnaire | 3-month comparison of groups: p=0.950 |
|  |  |  |  |  | HRQoL Total | AMS | Baseline comparison of groups: p=0.196  3-month comparison of groups: p=0.552  Change in experimental group before and after intervention: p=0.289 |
|  |  |  |  |  | Physical Symptoms | AMS | Baseline comparison of groups: p=0.217  3-month comparison of groups: p=0.295  Change in experimental group before and after intervention: p=0.838 |
|  |  |  |  |  | Psychological symptoms | AMS | Baseline comparison of groups: p=0.208  3-month comparison of groups: p=0.532  Change in experimental group before and after intervention: p=1.000 |
|  |  |  |  |  | Sexual symptoms | AMS | Baseline comparison of groups: p=0.411  3-month comparison of groups: p=0.823  Change in experimental group before and after intervention: p=0.036 |
|  |  |  |  |  | PSA | AMS | Baseline comparison of groups: p=0.299  3-month comparison of groups: p=0.747  Change in experimental group before and after intervention: p=0.000 |
| a) Yates et al. (2022)  b) Australia | a) Cohort study  b) Multiple health services in Australia | a) n=136  b) Prostate cancer  c) Mixed  d) Surgery, radiation therapy  Hormone  Chemo  Watchful waiting  e) Dyadic | a) TrueNTH care model including practical and social support, exercise and nutrition management, specialised support services, comorbidities management and partner/carer support  b) Nurse  c) 12 months  d) N/A | Baseline and 12 months post enrolment | Urinary incontinence | EPIC-26 | 12 months: p=0.18 |
|  |  |  |  |  | Urinary obstructive | EPIC-26 | 12 months: p=0.10 |
|  |  |  |  |  | Bowel | EPIC-26 | 12 months: p=0.68 |
|  |  |  |  |  | Sexual | EPIC-26 | 12 months: p=0.42 |
|  |  |  |  |  | Hormonal | EPIC-26 | 12 months: p=0.12 |
| a) Zhang et al. (2015)  b) USA | a) RCT  b) Hospital/ patient’s home | a) n=244 (n=81 in the support group, n=81 in the telephone group and n=82 in the control group  b) Prostate cancer  c) Not advanced  d) Surgery and radiation therapy  e) Not dyadic | a) Stay Dry program teaching PFME and self-management skills  b) BF technician, psychologist and CNS  c) 3 months  d) Control group received care as usual | Baseline, 3 months and 6 months post baseline | Daily leakage frequency | Diary data entry | Support:  3 months: p=0.019  6 months: p=0.555  Telephone:  3 months: p=0.001  6 months: p=0.069 |
|  |  |  |  |  | Leakage amount | 1-hour pad test | Support:  3 months: p=0.133  6 months: p=0.003  Telephone:  3 months: p=0.009  6 months: p=0.224  (Favouring support intervention: lower leakage amount) |
|  |  |  |  |  | Incontinence symptom severity | I-PSS | Support:  3 months: p=0.001  6 months: p=0.001  Telephone:  3 months: p=0.055  6 months: p=0.001  (Favouring support and telephone interventions: fewer incontinence symptoms) |
|  |  |  |  |  | Urinary function | UCLA-PCI | Support:  3 months: p=0.483  6 months: p=0.555  Telephone:  3 months: p=0.267  6 months: p=0.049  (Favouring telephone intervention) |
|  |  |  |  |  | Urinary function bother | 6-point item of symptom bother | Support:  3 months: p=0.878  6 months: p=0.181  Telephone:  3 months: p=0.262  6 months: p=0.009  (Favouring telephone intervention) |
|  |  |  |  |  | VAS rating in last 7 days | VAS | Support:  3 months: p=0.511  6 months: p=0.014  Telephone:  3 months: p=0.737  6 months: p=0.015  (Favouring support and telephone interventions: urinary incontinence  rated as less problematic) |
|  |  |  |  |  | VAS rating in last 4 weeks | VAS | Support:  3 months: p=0.237  6 months: p=0.0006  Telephone:  3 months: p=0.234  6 months: p=0.0024  (Favouring support and telephone interventions: urinary incontinence  rated as less problematic) |

***** p-value reported at the end of the study, rather than for each outcome measured.

****** p-value reported for statistically significant differences only.

**Abbreviations:**

6MWT=Six Minute Walking Test; AD= Androgen Deprivation; ADT=Androgen Deprivation Therapy; AEP=Accredited Exercise Physiologist; AMS=Aging Males Symptoms Scale; BF=Biofeedback; BIA=Bioelectrical Impedance Analyzer; BMI=Body Mass Index; BPI= Brief Pain Inventory-Short Form; CBSM=Cognitive Behavioural Stress Management; CES-D=Center for Epidemiologic Studies-Depression Scale; CNC=Clinical Nurse Coordinator; CNS=Clinical Nurse Specialist; CSMaleVS=International Continence Society Male questionnaire (voiding symptoms); DAS=Depression, Anxiety and Stress Scale; DBP=Diastolic blood pressure; EBRT=External Beam Radiation Therapy; EORTC QLQ-PR25=European Organisation for Research and Treatment of Cancer Quality-of-Life Scale; EPIC=Expanded Prostate Cancer Index Composite; FACT-F=Functional Assessment of Cancer Therapy-Fatigue; FACT-P=Functional Assessment of Cancer Therapy-Prostate; FOCUS= Family involvement, Optimistic attitude, Coping effectiveness, Uncertainty reduction, and Symptom management; HAD-A&D=Hospital Anxiety and Depression Scale; HEAC=Health Education Attention Condition; HP=Health Promotion; ICIQ SF=International Consultation on Incontinence Questionnaire-Short Form; ICIQ= International Consultation on Incontinence Questionnaire; ICSMaleIS=International Continence Society Male Questionnaire (incontinence symptoms); ICSMaleSF=International Continence Society Male Short Form questionnaire; ICSMaleVS= International Continence Society Male Questionnaire (voiding symptoms); IIEF=International Index of Erectile disfunction; IPSS=International Prostate Symptom Score; ISEL=Interpersonal Support Evaluation List; MAX-PC=Memorial Anxiety Scale for Prostate Cancer; Mini-MAC=Mini-mental Adjustment to Cancer Scale; MMSE=Mini Mental State Examination; MOS SF-12: Medical Outcomes Study 12-item Short Form Survey; MSD= Meso Scale Discovery; MFI= Multidimensional Fatigue Inventory; N/A= Not Applicable; NK= Natural Killer; NKCC=Natural Killer Cell Cytotoxicity; NR= Not Reported; OSQ=Omega Screening Questionnaire; PANAS=Positive and Negative Affect Schedule; PC=Prostate Cancer; PC-QoL=Prostate Cancer Quality of Life Instrument; PFME=Pelvic Floor Muscle Exercise; POMS=Profile of Mood States; PSA=Prostate Specific Antigen; PSS=Perceived Stress Scale; QoL=Quality of Life; RCT=Randomised Controlled Trial; SA=Supportive Attention; SBP=Systolic Blood Pressure; SC=Standard Care; SESCI=Self-Efficacy for Symptom Control Inventory; SF-36=36-Item Short Form Survey; SM=Stress Management; SMaRT=Symptom Management After Radiotherapy; STAI=Scale of the State Trait Anxiety Inventory; THE= Telephone Health Education; TIP-C= telephone interpersonal counselling; TUG=Timer Up&Go; UK=United Kingdom; USA=United States of America; VAS=Visual Analog Scale.
