# Supplementary material for: Supportive care interventions for men with urological cancers: a scoping review
Source: Support Care Cancer. 2023 Aug 21;31(9):530. doi: 10.1007/s00520-023-07984-0 (PMC10442278; doi:10.1007/s00520-023-07984-0)
Supplement: Supplementary file 2 — (DOCX 23 kb) [file 520_2023_7984_MOESM2_ESM.docx]

**Supplemental File 2.** Outcomes measured in the included studies (n=321 outcomes)

| **Physical health and wellbeing (n=61)** | **Exercise and physical health (n=36):**  Physical activity (n=3)  Physical function (n=2)  Systolic blood pressure (n=2)  Physical role (n=2)  Vitality (n=3)  Aerobic exercise tolerance (n=1)  Bench press (n=1)  Chair stand (n=1)  Completion of 400m walk (Cardiovascular and cardiorespiratory fitness variable) (n=1)  Diastolic blood pressure (n=1)  Energy expenditure (n=1)  Exercise healthy behaviour adherence (n=1)  Gait speed (n=1)  Hand grip strength (n=1)  Leg press (n=1)  Leg press, bench press, seated row, knee push ups, toe push ups, abdominal strength (all strength variables) (n=1)  Level of mobility (n=1)  Physical composite (n=1)  Physical functioning (n=3)  Physical health (n=1)  Physical performance (n=1)  Process of change for physical activity (n=1)  Resting heart rate (Cardiovascular and cardiorespiratory fitness variable) (n=1)  Role physical (n=1)  Six-minute walk (n=1)  Total exercise behaviour (n=1)  Walking endurance (n=1) |
| --- | --- |
|  | **Body weight and composition (n=15):**  Body mass index (n=4)  Fat mass (n=3)  Body weight (n=2)  Trunk mass (n=2)  Body fat (n=1)  Hip circumference (n=1)  Lean mass (n=1)  Waist circumference (n=1) |
|  | **Diet (n=6):**  Diet adherence (n=4)  Diet behaviour (n=2) |
|  | **Healthy behaviour (n=4):**  Bad habits healthy behaviour adherence (n=1)  Follow-up healthy behaviour adherence (n=1)  Healthy lifestyle (n=1)  Medication healthy behaviour adherence (n=1) |
| **Physical symptoms (n=72)** | **Urinary symptoms (n=33):**  Urinary incontinence (n=4)  Urinary irritation (n=2)  Urinary symptoms (n=3)  Urinary function (n=2)  Urinary Health – Irritative/Obstructive (n=2)  Daily leakage frequency (n=1)  Dynamic strength no of contractions during 60s (n=1)  Impact from incontinence on daily activities (n=1)  Impact from incontinence on QoL (n=1)  Incontinence aid (n=1)  Incontinence severity (n=1)  Incontinence symptom severity (n=1)  Incontinence symptoms (n=1)  Leakage amount (n=1)  Pad use (n=1)  Prostate cancer specific symptoms burden (n=1)  Static strength no. of seconds to hold one contraction (n=1)  Time to achieve continence after surgery (n=1)  Urinary (sum score) (n=1)  Urinary bother (n=1)  Urinary dysfunction (n=1)  Urinary function bother (n=1)  Urinary limitation (n=1)  Urinary obstructive (n=1)  Voiding symptoms (n=1) |
|  | **Gastrointestinal symptoms (n=19):**  Bowel symptoms (n=3)  Appetite loss (n=2)  Bowel dysfunction (n=2)  Constipation (n=2)  Diarrhoea (n=2)  Bowel Health (n=1)  Bloated abdomen (n=1)  Bowel (n=1)  Bowel bother (n=1)  Bowel function (n=1)  Bowel limitation (n=1)  Nausea and vomiting (n=1)  Patient perceived bother (eight bowel symptoms) (n=1) |
|  | **Pain (n=7):**  Bodily pain (n=3)  Pain (n=2)  Visual Analogue Scale rating in last 4 weeks (n=1)  Visual Analogue Scale rating in last 7 days (n=1) |
|  | **Fatigue (n=4):**  Fatigue (n=3)  Patient-reported fatigue (n=1) |
|  | **Hormonal symptoms (n=5)** |
|  | **Other symptoms (n=4):**  Physical symptoms (n=2)  Dyspnoea (n=1)  Insomnia (n=1) |
| **Psychological symptoms (n=29)** | **Depression (n=12):**  Depression (n=8)  Cancer knowledge on depression (n=1)  Chemotherapy on depression (n=1)  Prostate function on depression (n=1)  Social support on depression (n=1) |
|  | **Distress and stress (n=7):**  Psychological distress (n=2)  Distress (n=1)  Emotional distress (n=1)  Perceived stress (n=1)  Symptom distress (n=1)  Symptom distress on negative affect (n=1) |
|  | **Anxiety (n=8):**  Anxiety (n=5)  Anxiety preoccupation (n=2)  Cancer related anxiety (n=1) |
|  | **Mood (n=2)** |
| **Mental and emotional health (n=42)** | **Coping (n=20):**  Interpersonal coping (n=1)  Diversion coping (n=1)  Brief cope (n=1)  Fatalism (n=2)  Fighting spirit (n=2)  Helplessness (n=2)  Active coping (n=1)  Avoidant coping (n=1)  Cognitive avoidance (n=1)  Denial (n=1)  Diversion (n=1)  Endurance (n=1)  General coping techniques (n=1)  Hopelessness (n=1)  Regret (n=1)  Decision regrets (n=1)  Uncertainty (n=1) |
|  | **Affect (n=5):**  Positive affect (n=2)  Negative affect (n=1)  Symptom management on negative affect (n=1)  Prostate specific function on positive affect (n=1) |
|  | **Concerns and worries (n=4):**  Practical concerns (n=1)  Prostate specific antigen concern (n=1)  Fear of reoccurrence (n=1)  Health worry (n=1) |
|  | **Outlook (n=4):**  Cancer outlook (n=1)  Future outlook (n=1)  Outlook (n=1)  Positive outlook (n=1) |
|  | **Emotional health (n=4):**  Emotional concealment (n=1)  Emotional function (n=1)  Emotional role functioning (n=1)  Emotional role (n=1) |
|  | **Cognitive function (n=2):**  Cognition (n=1)  Cognitive function (n=1) |
|  | **Mental health (n=3)**  Mental health (n=2)  Mental composite (n=1) |
| **Sexuality and sexual health (n=27)** | Sexual function (n=4)  Sexual activity (n=3)  Sexual symptoms (n=3)  Sexual dysfunction (n=2)  Sexual health (n=1)  Sexual intimacy (n=2)  Mean non intercourse activities (n=1)  Overall use of treatments for sexual problems (n=1)  Satisfaction with sex life (n=1)  Sexual (n=1)  Sexual adjustment (n=1)  Sexual bother (n=1)  Sexual interest (n=1)  Sexual limitation (n=1)  Sexual relationship (n=1)  Sexual self-confidence (n=1)  Sexual supportive care needs (n=1)  Use of treatments for erectile function (n=1) |
| **Health perceptions and beliefs (n=8)** | General health (n=3)  General health perceptions (n=1)  Health beliefs (n=1)  Perceived exertion (n=1)  Perceived symptom improvement (n=1)  Positive attitude (n=1) |
| **Self-efficacy and confidence (n=13)** | Confidence in symptom self-management (n=1)  Self-esteem (n=2)  Self-efficacy for symptom control (n=1)  Self-efficacy in patient-physician interactions (n=1)  General confidence (n=1)  Self-efficacy (n=1)  Self-efficacy for coping with symptoms (n=1)  Self-efficacy for managing symptoms (n=1)  Self-efficacy for performing daily activities (n=1)  Self-efficacy for physical activity (n=1)  Self-efficacy for re-entry (n=1)  Stress management skills efficacy (n=1) |
| **Information and knowledge acquisition (n=18)** | Cancer knowledge on positive affect (n=1)  Disease information acquisition (n=1)  Disease knowledge mastery (n=1)  Hormone therapy disease knowledge mastery (n=1)  Information assistance (n=1)  Information satisfaction (n=1)  Less information (n=1)  Medical examination information acquisition (n=1)  More information (n=1)  Prostate cancer disease knowledge mastery (n=1)  Total disease knowledge mastery (n=1)  Total information acquisition (n=1)  Treatment information acquisition (n=1)  Video information (n=1)  Written information (n=1)  Other services information (n=1)  Medical places information (n=1)  Self-care information (n=1) |
| **Quality of life (n=14**) | Veternan quality of life (n=2)  Health-related quality of life (n=4)  Disease specific quality of life (n=1)  Mental quality of life (n=1)  Overall quality of life (n=1)  Physical quality of life (n=1)  Quality of life-mental (n=1)  Quality of life-physical (n=1)  Symptom related quality of life (n=1)  Global health status and QoL (n=1) |
| **Social support and relationships (n=19)** | Social functioning (n=2)  Appraisal (n=1)  Belonging (n=1)  Domestic (n=1)  Extended family (n=1)  Marital affection (n=1)  Marital interactions (n=1)  Medical interactions (n=1)  Social environment (n=1)  Social function (n=1)  Social support (n=1)  Social support on positive affect (n=1)  Social support-family (n=1)  Social wellbeing (n=1)  Spiritual wellbeing (n=1)  Tangible (n=1)  Dyadic cohesion (n=1)  Dyadic satisfaction (n=1) |
| **Others (n=18)** | Cancer control (n=2)  Patient satisfaction (n=2)  Prostate specific antigen (n=2)  Appraisal of illness (n=1)  Communication (n=1)  Digital evaluation (n=1)  Financial difficulties (n=1)  Healthcare orientation (n=1)  Informed decision (n=1)  Planning (n=1)  Resumption of normal activities (n=1)  Return to work (n=1)  Self-decision making (n=1)  Self-decompression (n=1)  Vocational environment (n=1) |
