# Supplementary material for: Supportive care interventions for men with urological cancers: a scoping review
Source: Support Care Cancer. 2023 Aug 21;31(9):530. doi: 10.1007/s00520-023-07984-0 (PMC10442278; doi:10.1007/s00520-023-07984-0)
Supplement: Supplementary file 3 — (DOCX 18 kb) [file 520_2023_7984_MOESM3_ESM.docx]

**Supplemental File 3.** Instruments and measures used in the included studies (n=112 instruments and measures).

| **Instrument/measure** | **Number of studies** |
| --- | --- |
| EPIC | n=6 |
| CES-D | n=5 |
| SF-36 | n=4 |
| EORTC QLQ-PR25 | n=3 |
| PANAS | n=3 |
| 6MWT | n=3 |
| EORTC QLQ-C30 | n=2 |
| EPIC-26 | n=2 |
| SF-12 | n=2 |
| Kg  Kg, reps, sec  Kg/m  Kcal/kg/day | n=2 |
| MAX-PC | n=2 |
| MFI | n=2 |
| cm | n=2 |
| BPI | n=2 |
| STAI | n=2 |
| ISEL | n=2 |
| FACT-G (n=2) | n=2 |
| HAD-A | n=2 |
| PC QoL scale | n=1 |
| 14-item author-constructed 11-point scale | n=1 |
| 28-item Brief Cope instrument | n=1 |
| 5-item scale | n=1 |
| EORTC QLQ-INFO25 | n=1 |
| EORTC QLQ-30 | n=1 |
| EPIC-UI | n=1 |
| 50 item EPIC | n=1 |
| ICIQ | n=1 |
| ICIQ-SF | n=1 |
| Psychological adjustment to illness scale | n=1 |
| DAS-21 | n=1 |
| DAS | n=1 |
| Self-designed healthy behavior adherence questionnaire | n=1 |
| Self-designed disease knowledge questionnaire | n=1 |
| AMS | n=1 |
| PROMIS | n=1 |
| Mini-MAC | n=1 |
| MOS | n=1 |
| MOS SF-12 | n=1 |
| Mental adjustment to cancer scale | n=1 |
| 1-RM protocol | n=1 |
| DXA | n=1 |
| The Measuring Patients’ Perception of the Outcomes of Treatment for Early PC | n=1 |
| Strategies Used by People to Promote Health | n=1 |
| IPSS | n=1 |
| IPSS QoL | n=1 |
| SESCI | n=1 |
| FACT-F (n=1) | n=1 |
| FACT-P (n=1) | n=1 |
| 5-item marital interactions scale | n=1 |
| 5 items medical interactions scale | n=1 |
| 28-item Brief Coping Orientations to Problems Experienced scale | n=1 |
| 12-item practical concerns scale | n=1 |
| Hospital anxiety and depression scale | n=1 |
| ICSmaleVS | n=1 |
| IIEF | n=1 |
| mmHg | n=1 |
| PEPPI | n=1 |
| PSS | n=1 |
| PSS-FA | n=1 |
| VAS | n=1 |
| Rotterdam symptom checklist | n=1 |
| Rieker sexual adjustment scale | n=1 |
| 16-item Symptom Scale of the OSQ | n=1 |
| 17-item Lewis Cancer Self-efficacy Scale | n=1 |
| 1-hour pad test | n=1 |
| 20-item Beck Hopelessness Scale | n=1 |
| 27-item Appraisal of Illness | n=1 |
| 28-item Mishel Uncertainty in Illness Scale | n=1 |
| 32-item Lewis Mutuality and Interpersonal Sensitivity Scale | n=1 |
| 3-d diet diaries analysed using NetWisp v.3.0 | n=1 |
| 3-item diversion subscale | n=1 |
| 3-item planning subscale | n=1 |
| 3-item positive subscale | n=1 |
| 6-point item of symptom bother | n=1 |
| 7-item interpersonal coping scale | n=1 |
| Bioelectrical impedance analyzer | n=1 |
| Borg Scale | n=1 |
| bpm | n=1 |
| Brannon masculinity scale | n=1 |
| British Hypertension Society Guidance | n=1 |
| Cancer Coping Questionnaire | n=1 |
| CHAMPS questionnaire | n=1 |
| days/week active for >30 min | n=1 |
| Decisional Balance for Physical Activity Questionnaire | n=1 |
| Diary data entry | n=1 |
| Distress Thermometer | n=1 |
| FFQ | n=1 |
| Geneva BIA formula | n=1 |
| GISEQ | n=1 |
| Godin Leisure Score Index | n=1 |
| Height (wall-mounted stadiometer) and weight (Weylux beam balance scales) | n=1 |
| MMSE | n=1 |
| MOCS | n=1 |
| Physical activity self-efficacy questionnaire | n=1 |
| POMS | n=1 |
| PPB | n=1 |
| Processes of Change for Physical Activity Questionnaire | n=1 |
| Satisfaction with Consultation Scale | n=1 |
| Schover scale | n=1 |
| Sec | n=1 |
| Social wellbeing scale | n=1 |
| Spiritual wellbeing subscale | n=1 |
| Supportive Care Needs Survey | n=1 |
| Symptom-limited graded exercise test | n=1 |
| The Masculine Self‐Esteem Scale | n=1 |
| TUG | n=1 |
| UCLA-PCI | n=1 |
| Weight divided by height | n=1 |
| VR-12 | n=1 |
